# Adult weight change across the life course and cardiovascular disease prevalence and mortality: a population-based study

**DOI:** 10.3389/fpubh.2026.1879396

**Published:** 2026-07-02

**Authors:** Ju Li, Yajie Zhao, Xueqiang Wu, Ling Tao

**Affiliations:** 1 Xinyang Vocational and Technical College, Xinyang, Henan, China; 2 Henan Engineering Research Center for Digital & Intelligent Diagnosis and Treatment of Hematology & Oncology, Xinyang, Henan, China

**Keywords:** cardiovascular disease, cohort study, mortality, prevalence, weight change

## Abstract

**Background:**

Associations between long-term adult weight change and cardiovascular disease (CVD) risk remain incompletely understood, especially regarding life-stage-specific patterns. We examined how weight-change trajectories across adulthood relate to CVD prevalence and mortality in a representative population.

**Methods:**

We analyzed 35,998 adults aged ≥ 40 years in multiple townships or sub-districts, Xinyang City, Henan Province, China from 2010 to 2024. BMI at age 25, 10 years before baseline, and baseline was used to classify patterns: stable normal weight, maximum overweight, obese-to-non-obese, non-obese-to-obese, and stable obesity. Multivariable logistic regression estimated odds ratios (ORs) for CVD prevalence and subtypes; Cox models estimated hazard ratios (HRs) for CVD mortality, accounting for survey design.

**Results:**

At baseline, 6,112 participants had prevalent CVD. Over a median follow-up of 9.2 years (310,647 person-years), 2,314 participants died from CVD-related causes. From young to mid-adulthood, stable obesity was associated with higher odds of overall CVD (OR 1.92; 95% CI 1.38–2.65) and heart failure (OR 3.49; 95% CI 2.41–5.06). Transitioning from non-obesity to obesity also increased CVD risk. From mid to late adulthood, transitioning from obesity to non-obesity was associated with higher CVD prevalence (OR 2.00; 95% CI 1.61–2.48) and CVD mortality (HR 1.58; 95% CI 1.22–2.05). Large weight gain (≥ 20 kg) increased CVD risk, whereas later-life weight loss was associated with elevated CVD mortality.

**Conclusion:**

Adult weight change relates to CVD risk in life-stage-specific and heterogeneous ways. Maintaining normal weight from early adulthood and preventing excessive long-term weight gain may reduce CVD risk. Considering the timing of body weight changes across the adult lifespan is of great public health significance for cardiovascular risk assessment.

## 1 Introduction

CVD remains the leading cause of morbidity and mortality globally, accounting for a substantial proportion of premature mortality and long-term disability despite continuous advances in preventive and therapeutic strategies (1, 2). Excess adiposity and obesity have been increasingly recognized as major modifiable risk factors for CVD, and weight management has thus emerged as a central component of contemporary cardiovascular prevention guidelines (3). Most epidemiological studies (4–6), however, have relied on BMI measured at a single time point to quantify adiposity-associated risk, a static approach that fails to adequately capture the dynamic fluctuations in body weight across adulthood.

In recent years, mounting attention has been devoted to weight change and long-term weight trajectories as indicators that may be more informative of cardiovascular risk than static BMI measurements (7, 8). Several longitudinal studies have established that persistent obesity or substantial weight gain from early adulthood confers elevated risks of coronary heart disease, congestive heart failure, and stroke (9–11). In contrast, evidence pertaining to weight loss has remained far less consistent. Although intentional weight reduction has been linked to improvements in metabolic risk factors (12, 13), observational studies—particularly those conducted in older populations—have frequently reported positive associations between weight loss and adverse cardiovascular outcomes or mortality (5, 14, 15). These discrepant findings may be ascribed to variations in study design, the timing of weight change, baseline health status, and the inability to discriminate between intentional and unintentional weight loss (16, 17).

Notably, existing studies evaluating adult weight change and CVD have several key limitations. Many have focused on restricted age ranges or short follow-up periods, employed heterogeneous or simplified weight change definitions, or examined a single cardiovascular outcome (18–22). Moreover, relatively few population-based studies have concurrently assessed associations of weight change patterns across multiple adult stages with both CVD prevalence and CVD-related mortality. Whether the cardiovascular implications of weight change differ across life stages—from early to middle adulthood versus later adulthood—and whether these associations vary by age or sex therefore remains incompletely understood.

This study is guided by the life course epidemiology framework, and based on existing literature and the above theoretical basis, we proposed research hypotheses: (1) Unreasonable weight gain during a certain period of an adult’s life may be associated with the risk of developing and dying from CVD. (2) the associations between weight change patterns and CVD outcomes differ by age and gender.

In this study, we used data from multiple towns or sub-districts in Xinyang City 2010–2024 to systematically examine associations between long-term weight change patterns across adulthood and cardiovascular disease prevalence and mortality in a representative sample of adults. Weight change patterns were characterized across three adulthood intervals spanning from early adulthood to later life. We further evaluated potential heterogeneity by age and sex. By jointly assessing life-stage-specific weight change patterns in relation to both CVD prevalence and mortality, this study aimed to provide a comprehensive evaluation of the long-term cardiovascular implications of adult weight dynamics.

## 2 Methods

### 2.1 Study design and participant selection

This study utilized data from multiple townships or sub-districts in Xinyang City. It was a population-based study comprising a cross-sectional assessment of baseline health status and prospective follow-up analyses of subsequent health outcomes among community-dwelling adults.

The survey adopted a multi-stage stratified cluster sampling method: firstly, we randomly selected representative townships and urban sub-districts across Xinyang City; secondly, communities or villages were sampled within the selected sites; finally, all eligible residents aged 40 years and older in the sampled communities were invited to participate in the baseline survey. A total of 68,569 residents were initially enrolled in this baseline survey. The survey employed a complex multi-stage probability sampling design and conducted additional targeted sampling of key demographic groups to enhance sample representativeness. Xinyang City is located in central China, with a mixed population of urban and rural residents. The demographic structure, socioeconomic status, lifestyle habits (diet, smoking, alcohol use, physical activity) and chronic disease prevalence of local adults are highly consistent with the general middle-aged and older adults in inland central China. Therefore, our sample derived from Xinyang City is representative of community-dwelling adults in this region, and the study findings can be generalized to similar populations across central China.

**Figure 1:**
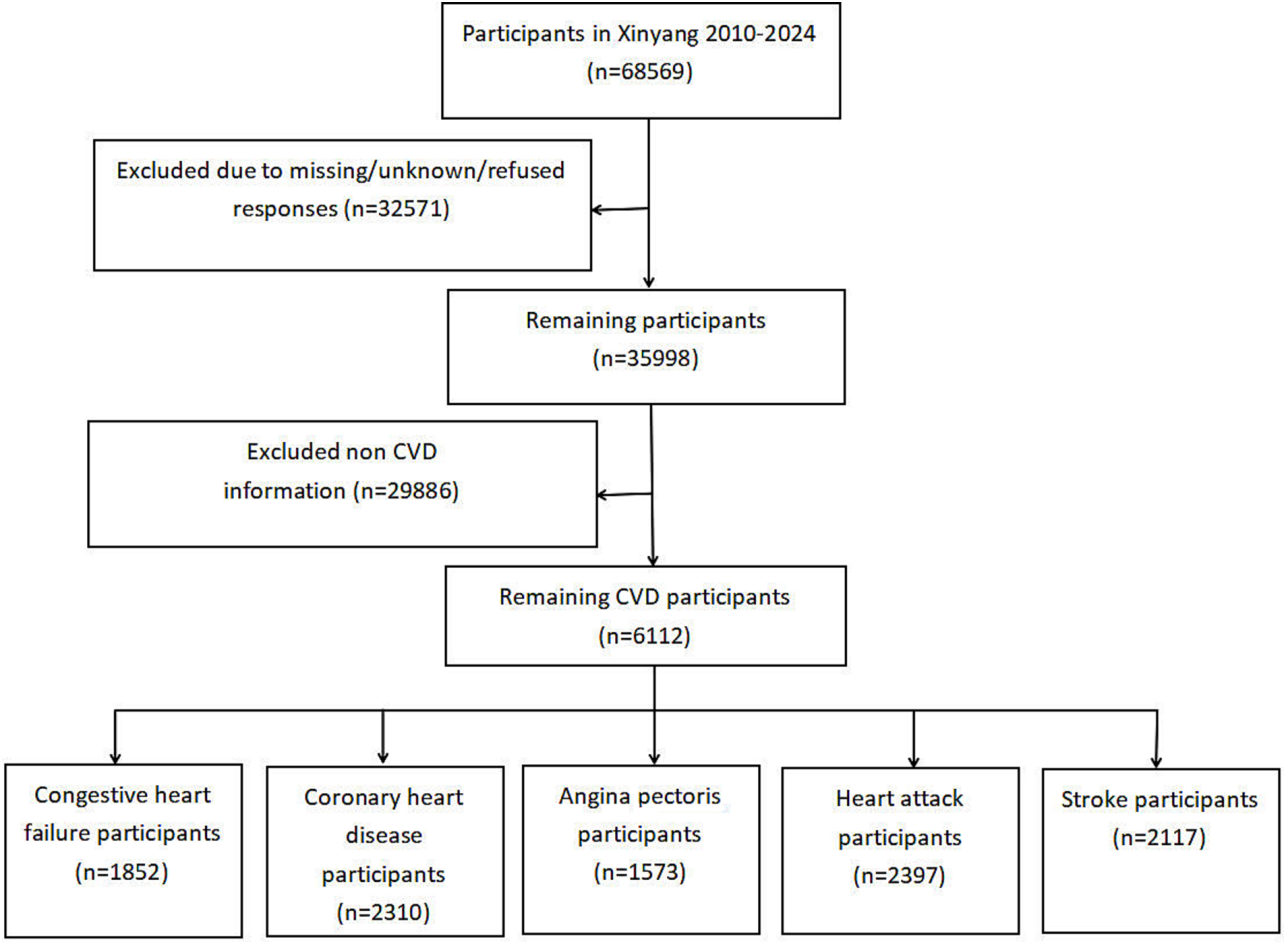
Flow chart for selecting CVD cases in Xinyang cohort from 2010 to 2024.

For the present analysis, adults aged ≥40 years at baseline who had available BMI data at age 25 years, 10 years before baseline, and at baseline were eligible for inclusion. Participants with missing weight history or CVD outcome data were excluded. The selection of participants for analyses of prevalent CVD is illustrated in Figure 1. A total of 68,569 participants were initially considered. Participants with missing, unknown, or refused information on key study variables were excluded (*n* = 32,571). The remaining analytic sample consisted of 35,998 participants with complete data on body mass index history and CVD status.

Among these participants, prevalent CVD was defined as a self-reported physician diagnosis of congestive heart failure, coronary heart disease, angina pectoris, myocardial infarction, or stroke. A total of 6,112 participants were classified as having prevalent CVD, whereas 29,886 participants without CVD were excluded from subtype-specific analyses. Among participants with prevalent CVD, the numbers of cases for specific CVD subtypes were as follows: congestive heart failure (*n* = 1,852).

coronary heart disease (*n* = 2,310), angina pectoris (*n* = 1,573), myocardial infarction (*n* = 2,397), and stroke (*n* = 2,117).

### 2.2 Assessment of body weight and weight change patterns

Body weight and height at baseline were measured by trained personnel using standardized protocols during the mobile examination center visit. BMI was calculated as weight in kilograms divided by height in meters squared (kg/m^2^). BMI at age 25 years and 10 years before baseline was obtained from standardized self-reported questionnaires.

Weight change patterns across adulthood were defined on the basis of BMI categories at the three time points (age 25 years, 10 years before baseline, and baseline). Participants were classified into five mutually exclusive weight change patterns: (1) stable normal weight (BMI < 24.0 kg/m^2^ at all assessed time points); (2) obesity); (3) obese to non-obese (BMI ≥ 28.0 kg/m^2^ at an earlier time point and < 28.0 kg/m^2^ at a later time point); (4) non-obese to obese (BMI < 28.0 kg/m^2^ at an earlier time point and ≥ 28.0 kg/m^2^ at a later time point); and (5) stable obesity (BMI ≥ 28.0 kg/m^2^ at all assessed time points). Weight change patterns were evaluated across three adulthood intervals: from age 25 years to 10 years before baseline, from 10 years before baseline to baseline, and across the entire adulthood period.

### 2.3 Ascertainment of cardiovascular disease outcomes

Prevalent CVD was ascertained using standardized self-reported questionnaires administered during interviews. Participants were asked whether a physician or other health professional had ever diagnosed them with congestive heart failure, coronary heart disease, angina pectoris, myocardial infarction, or stroke. Participants reporting at least one of these conditions were classified as having prevalent CVD.

**Figure 2:**
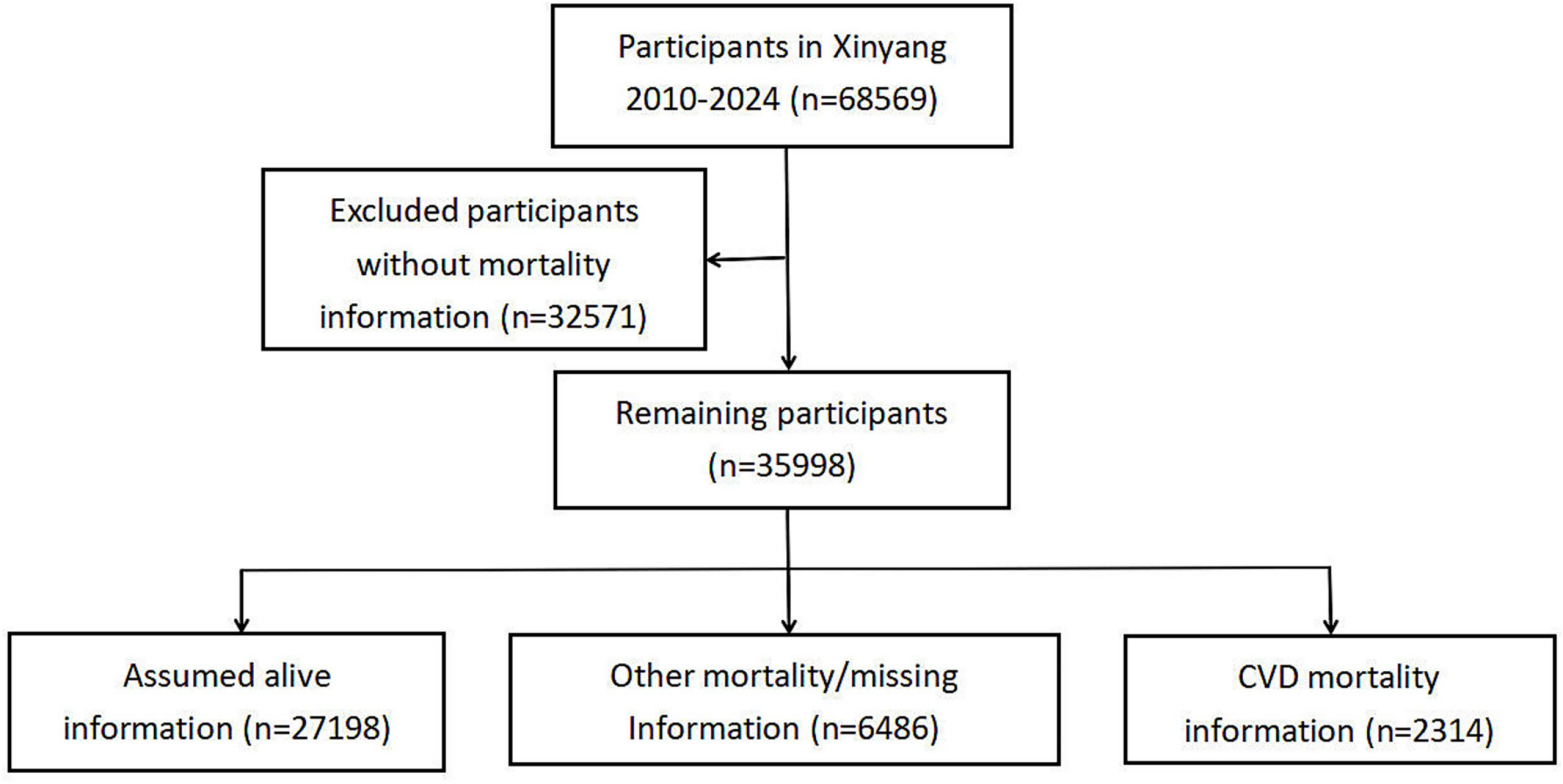
Flow chart for selecting CVD death cases in Xinyang cohort from 2010 to 2024.

The definitive follow-up date was uniformly set as December 31, 2024. The selection of participants for the CVD mortality analysis is illustrated in Figure 2. Among the 68,569 participants initially assessed, those without mortality information were excluded (*n* = 32,571). The remaining 35,998 participants were included in the mortality analysis. During follow-up, 2,314 participants died from CVD-related causes, 27,198 were classified as alive at follow-up end, and 6,486 with missing cause-of-death information were excluded from CVD mortality analyses. Person-years of follow-up were calculated from the baseline examination date to the date of death or the end of follow-up, whichever occurred first.

### 2.4 Covariates

Covariates were selected *a priori* on the basis of established associations with both body weight and cardiovascular outcomes. Demographic variables included age (years), sex (male or female), race and ethnicity (Han, or other races), educational attainment (below high school or high school or above), marital status (married or living with partner versus other), and annual family income. Lifestyle factors included smoking status (current smoker versus non-smoker), alcohol consumption (yes or no), and moderate physical activity (yes or no). Clinical covariates included baseline BMI (kg/m^2^), self-rated general health status, family history of heart attack, and family history of diabetes.

### 2.5 Statistical analysis

Baseline characteristics were summarized according to weight change patterns using weighted means with 95% confidence intervals (CIs) for continuous variables and weighted proportions for categorical variables. Differences across groups were assessed using analysis of variance for continuous variables and the Rao-Scott *χ*^2^ test for categorical variables.

Multivariable logistic regression models were used to estimate odds ratios (ORs) and 95% CIs for associations between weight change patterns and prevalent CVD as well as major CVD subtypes. Fine-Gray subdistribution hazard models were applied to estimate hazard ratios (HRs) and 95% CIs for CVD-related mortality. Sequential models were constructed with progressive adjustment for demographic, lifestyle, and clinical covariates.

All models accounted for the complex survey design by incorporating sampling weights, strata, and primary sampling units. Subgroup analyses were conducted by age (≤65 versus >65 years) and sex, and potential effect modification was assessed by including interaction terms. Proportional hazards assumptions were evaluated using Schoenfeld residuals. All statistical analyses were performed using SAS software version 9.4 (SAS Institute, Cary, NC). Statistical significance was defined as a two-sided *p* value < 0.05.

### 2.6 Ethics statement

This study was conducted according to the guidelines laid down in the Declaration of Helsinki and all procedures involving human subjects were approved by the Biomedical Ethics Committee of Shanghai University of Medicine & Health Sciences (26-PH-H06). Written informed consent was obtained from all participant.

## 3 Results

### 3.1 Baseline characteristics of the study population

The baseline characteristics of the study population according to weight change patterns across adulthood are presented in Table 1. Among the 35,998 participants included in the analysis, the mean age at baseline was 57.5 years, and 52.8% were women. Overall, 30.8% of participants maintained stable normal weight from age 25 to baseline, 36.5% experienced maximum overweight, 28.9% transitioned from non-obesity to obesity, 2.7% remained stably obese, and 1.0% transitioned from obesity to non-obesity. For age 25 years to 10 years before baseline and from 10 years before baseline to baseline, the corresponding data are presented in Supplementary Tables S1, S2 respective.

**Table 1:** Baseline characteristics of study population according to weight change patterns from age 25 years to baseline.

Characteristics^b^	Total	Weight change patterns from age 25 years to baseline^a^					*p* value^c^
		Stable normal	Maximum overweight	Obese to non-obese	Non-obese to obese	Stable obese	
Study population (*n*/%)	35,998	10,089 (30.80)	13,149 (36.53)	373 (1.04)	10,407 (28.91)	980 (2.72)	–
Age means (95% CI), years	57.53 (57.27–57.78)	58.21 (57.82–58.60)	57.93 (57.59–58.26)	62.26 (61.05–63.47)	56.00 (55.64–56.36)	59.58 (59.01–60.16)	<0.01
Sex							
Male	17,541 (47.17)	4,638 (36.22)	7,620 (57.01)	222 (57.28)	4,577 (45.70)	484 (51.08)	<0.01
Female	18,457 (52.83)	6,451 (63.78)	5,529 (42.99)	151 (42.72)	5,830 (54.30)	496 (48.92)	
BMI means (95% CI), kg/m^2^							
At the age of 25	21.03 (20.94–21.11)	20.58 (20.52–20.64)	21.77 (21.68–21.85)	28.96 (28.67–29.24)	21.82 (21.70–21.95)	28.58 (28.14–29.03)	<0.01
At 10 years prior to baseline	25.04 (24.93–25.16)	21.44 (21.37–21.51)	24.38 (24.29–24.47)	26.37 (26.06–26.68)	27.29 (27.13–27.44)	30.95 (30.12–31.38)	<0.01
At baseline survey	27.39 (27.27–27.51)	22.29 (22.15–22.37)	25.20 (25.16–25.34)	24.73 (24.32–25.14)	29.96 (29.84–30.07)	31.12 (30.41–31.82)	<0.01
Absolute weight change mean (95% CI), kg	10.84 (10.59–11.09)	3.30 (3.10–3.59)	8.57 (8.41–8.63)	−15.69 (−17.70--13.58)	23.55 (23.11–23.88)	9.28 (7.64–11.82)	<0.01
Race or ethnicity (*n*/%)							
Han	35,711(99.20)	14,968 (98.93)	10,999 (99.41)	294 (98.00)	8,633 (99.87)	817 (95.00)	<0.01
Others	287 (0.80)	162 (1.07)	65 (0.59)	6(2.00)	11 (0.13)	43 (5.00)	
Marital status (*n*/%)							
Married or living with partner	21,869 (67.36)	6,428 (64.99)	8,489 (70.99)	204 (60.48)	6,212 (65.85)	536 (63.09)	<0.01
Others	13,751 (32.64)	4,515 (35.01)	4,538 (29.01)	165 (39.52)	4,098 (34.15)	435 (36.91)	
Annual family income (*n*/%)							
Less than ¥9,999	12,334 (24.27)	3,995 (26.28)	4,258 (21.77)	166 (33.99)	3,513 (24.57)	402 (29.71)	<0.01
¥10,000 to ¥19,999	14,296 (42.93)	4,021 (39.99)	5,450 (43.96)	142 (39.61)	4,312 (44.65)	371 (44.19)	
¥20,000 and over	7,353 (32.80)	2,307 (33.73)	2,783 (34.27)	45 (26.40)	2061 (30.78)	157 (26.10)	
Educational levels (*n*/%)							
Below high school	34,366 (95.72)	10,418 (94.48)	12,523 (97.26)	404 (92.14)	10,107 (95.16)	914 (96.19)	<0.01
High school or above	1,537 (4.28)	609 (5.52)	353 (2.74)	25 (5.83)	514 (4.84)	36 (3.81)	
Alcohol drinking (*n*/%)							
Yes	20,210 (75.93)	5,702 (74.96)	7,789 (78.94)	226 (79.03)	5,929 (73.59)	564 (71.18)	<0.01
No	8,460 (24.07)	2,681 (25.04)	2,732 (21.06)	76 (20.97)	2,703 (26.41)	268 (28.82)	
Smoking status (*n*/%)							
Yes	17,738 (49.35)	5,303 (48.50)	6,652 (49.77)	215 (59.00)	5,069 (49.49)	499 (49.26)	0.13
No	18,212 (50.65)	5,753 (51.50)	6,488 (50.23)	158 (41.00)	5,333 (50.51)	480 (50.74)	
Moderate activity (*n*/%)							
Yes	12,889 (43.20)	3,715 (42.33)	4,937 (45.03)	110 (38.43)	3,788 (42.36)	339 (38.22)	<0.01
No	22,279 (56.80)	7,038 (57.67)	7,981 (54.97)	246 (61.57)	6,398 (57.64)	616 (61.78)	
General health condition (*n*/%)							
Very good to excellent	12,756 (44.96)	4,594 (53.12)	5,091 (48.71)	112 (44.60)	2,767 (33.57)	192 (24.60)	<0.01
Good	12,666 (33.96)	4,693 (33.51)	4,693 (33.51)	121 (28.55)	3,926 (39.56)	372 (41.99)	
Poor to fair	10,550 (21.08)	2,932 (18.28)	3,358 (17.79)	140 (26.85)	3,704 (26.87)	416 (33.41)	
Family history of heart attack (*n*/%)							
Yes	4,227 (18.08)	1,109 (16.26)	1,441 (17.41)	54 (19.54)	1,472 (20.46)	151 (20.17)	<0.01
No	21,909 (81.92)	6,690 (83.74)	7,950 (82.59)	231 (80.46)	6,421 (79.54)	617 (79.83)	
Family history of diabetes (*n*/%)							
Yes	14,543 (48.40)	3,579 (40.39)	5,135 (47.27)	176 (51.75)	5,108 (56.37)	545 (60.82)	<0.01
No	14,238 (51.60)	4,923 (59.61)	5,250 (52.73)	140 (48.25)	3,621 (48.25)	304 (39.18)	
Cardiovascular diseases (*n*/%)							
Yes	6,112 (13.35)	1,617 (10.57)	2,219 (13.27)	104 (22.17)	1919 (15.33)	253 (22.02)	<0.01
No	29,886 (86.65)	9,472 (89.43)	10,930 (86.73)	269 (77.83)	8,488 (84.67)	727 (77.98)	
Congestive heart failure (*n*/%)							
Yes	1852 (3.71)	441 (2.69)	600 (3.19)	43 (7.86)	661 (4.88)	107 (8.69)	<0.01
No	34,146 (96.29)	10,648 (97.31)	12,549 (96.81)	330 (92.14)	9,746 (95.12)	873 (91.31)	
Coronary heart disease (*n*/%)							
Yes	2,310 (5.42)	592 (4.10)	898 (5.69)	46 (9.13)	677 (5.91)	97 (10.46)	<0.01
No	33,688 (94.58)	10,497 (95.90)	12,251 (94.31)	327 (90.87)	9,730 (94.09)	883 (89.54)	
Angina pectoris (*n*/%)							
Yes	1,573 (3.76)	378 (2.71)	559 (3.59)	22 (4.22)	542 (4.74)	72 (7.41)	<0.01
No	34,425 (96.24)	10,711 (97.29)	12,590 (96.41)	351 (95.78)	9,865 (95.26)	908 (92.59)	
Heart attack (*n*/%)							
Yes	2,397 (5.30)	612 (3.95)	891 (5.43)	58 (12.29)	727 (5.92)	109 (10.33)	<0.01
No	33,601 (94.70)	10,477 (96.05)	12,258 (94.57)	315 (87.71)	9,680 (94.08)	871 (89.67)	
Stroke (*n*/%)							
Yes	2,117 (4.30)	624 (3.92)	744 (4.06)	37 (6.98)	634 (4.77)	78 (6.23)	<0.01
No	33,881 (95.70)	10,465 (96.08)	12,405 (95.94)	336 (93.02)	9,773 (95.23)	902 (93.77)	

95% CI, 95% Confidence Interval; BMI, Body Mass Index.

a All estimates accounted for complex survey designs.

b Regarding the information on baseline educational level, marital status, family annual income, drinking habits, overall health condition, moderate exercise status, smoking habits, family history of heart attack and Family history of diabetes, there were 95, 378, 2015, 7,328, 26, 830, 48, 9,862, and 7,217 participants who had missing information, respectively.

c The *p*-values for categorical variables are calculated using the Rao-Scott chi-square test, which is a modified version of the pearson chi-square test. The P-values for continuous variables are calculated through variance analysis that takes into account the adjustment of sampling weights.

Participants with stable obesity or transition from non-obesity to obesity had higher BMI at all assessed time points and exhibited less favorable cardiometabolic profiles compared with those maintaining stable normal weight. In contrast, participants who transitioned from obesity to non-obesity had substantially higher BMI at age 25 but lower BMI at baseline. The prevalence of CVD and major CVD subtypes, including congestive heart failure, coronary heart disease, myocardial infarction, and stroke, differed significantly across weight change patterns (all *p* < 0.01).

### 3.2 Weight change patterns and prevalence of cardiovascular disease

Associations between weight change patterns across adulthood and prevalent CVD are shown in Table 2. Compared with participants maintaining stable normal weight, those with stable obesity had higher odds of prevalent CVD across adulthood, even after full multivariable adjustment (OR, 1.91; 95% CI, 1.39–2.64). Transition from non-obesity to obesity was also associated with increased odds of prevalent CVD (OR, 1.71; 95% CI, 1.45–2.00).

**Table 2:** Odds ratio (95% CIs) of CVD with weight change patterns.^a^

	Weight change patterns				
	Stable normal	Maximum overweight	Obese to non-obese	Non-obese to obese	Stable obese
From age 25 years to 10 years before baseline					
Prevalence of cardiovascular diseases					
Model 1	1.00	1.31 (1.18–1.45)	1.82 (1.15–2.86)	1.90 (1.71–2.12)	2.48 (1.95–3.14)
Model 2	1.00	1.32 (1.16–1.51)	1.42 (0.85–2.37)	1.90 (1.64–2.20)	2.40 (1.76–3.28)
Model 3	1.00	1.24 (1.08–1.43)	1.27 (0.74–2.17)	1.66 (1.42–1.94)	1.92 (1.38–2.65)
Prevalence of congestive heart failure					
Model 1	1.00	1.34 (1.12–1.60)	2.92 (1.69–5.05)	2.50 (2.09–2.98)	3.73 (2.77–5.02)
Model 2	1.00	1.36 (1.05–1.77)	2.98 (1.56–5.72)	2.60 (2.05–3.31)	4.22 (2.93–6.08)
Model 3	1.00	1.28 (0.97–1.70)	2.67 (1.41–5.06)	2.32 (1.81–2.99)	3.49 (2.41–5.06)
Prevalence of coronary heart disease					
Model 1	1.00	1.28 (1.10–1.49)	2.13 (1.17–3.88)	1.71 (1.43–2.03)	2.60 (1.88–3.57)
Model 2	1.00	1.21 (0.97–1.50)	2.11 (1.03–4.31)	1.52 (1.20–1.93)	2.34 (1.49–3.70)
Model 3	1.00	1.12 (0.89–1.40)	2.10 (1.03–4.29)	1.33 (1.04–1.69)	1.94 (1.22–3.09)
Prevalence of angina pectoris					
Model 1	1.00	1.33 (1.09–1.62)	1.58 (0.68–3.65)	1.73 (1.43–2.07)	2.52 (1.72–3.72)
Model 2	1.00	1.24 (0.93–1.66)	1.77 (0.76–4.12)	1.64 (1.27–2.11)	2.39 (1.38–4.15)
Model 3	1.00	1.12 (0.84–1.51)	1.70 (0.72–3.98)	1.37 (1.07–1.76)	1.79 (1.03–3.13)
Prevalence of heart attack					
Model 1	1.00	1.27 (1.08–1.48)	1.55 (0.86–2.79)	1.72 (1.49–1.98)	2.92 (2.14–4.00)
Model 2	1.00	1.21 (0.98–1.49)	1.36 (0.63–2.92)	1.62 (1.31–2.00)	2.86 (1.83–4.45)
Model 3	1.00	1.15 (0.92–1.43)	1.18 (0.52–2.69)	1.44 (1.16–1.79)	2.37 (1.52–3.69)
Prevalence of stroke					
Model 1	1.00	1.21 (1.05–1.39)	1.86 (0.94–3.65)	1.77 (1.51–2.07)	1.85 (1.37–2.50)
Model 2	1.00	1.22 (1.00–1.48)	1.30 (0.58–2.93)	1.70 (1.36–2.12)	1.87 (1.29–2.70)
Model 3	1.00	1.12 (0.92–1.36)	1.20 (0.53–2.74)	1.46 (1.16–1.84)	1.48 (1.03–2.13)
From age 25 years to baseline					
Prevalence of cardiovascular diseases					
Model 1	1.00	1.31 (1.20–1.43)	2.03 (1.42–2.90)	2.00 (1.80–2.22)	2.69 (2.10–3.43)
Model 2	1.00	1.33 (1.18–1.50)	1.97 (1.20–3.24)	1.99 (1.70–2.32)	2.52 (1.84–3.44)
Model 3	1.00	1.30 (1.16–1.47)	2.03 (1.21–3.40)	1.71 (1.45–2.00)	1.91 (1.39–2.64)
Prevalence of congestive heart failure					
Model 1	1.00	1.21 (1.03–1.43)	2.56 (1.71–3.82)	2.40 (2.01–2.88)	3.99 (2.93–5.43)
Model 2	1.00	1.25 (0.99–1.57)	2.11 (1.25–3.56)	2.47 (1.94–3.14)	4.70 (3.21–6.88)
Model 3	1.00	1.23 (0.99–1.52)	2.08 (1.24–3.48)	2.14 (1.67–2.74)	3.74 (2.55–5.47)
Prevalence of coronary heart disease					
Model 1	1.00	1.32 (1.16–1.50)	1.87 (1.19–2.94)	1.87 (1.57–2.22)	3.03 (2.13–4.31)
Model 2	1.00	1.21 (1.01–1.45)	1.92 (1.14–3.22)	1.70 (1.31–2.21)	2.65 (1.62–4.36)
Model 3	1.00	1.15 (0.96–1.37)	2.02 (1.20–3.39)	1.45 (1.11–1.88)	2.11 (1.27–3.50)
Prevalence of angina pectoris					
Model 1	1.00	1.33 (1.13–1.57)	1.37 (0.76–2.48)	2.20 (1.85–2.61)	3.15 (2.08–4.75)
Model 2	1.00	1.24 (0.96–1.61)	1.54 (0.77–3.07)	1.90 (1.48–2.46)	2.84 (1.56–5.18)
Model 3	1.00	1.15 (0.89–1.48)	1.58 (0.79–3.17)	1.55 (1.19–2.01)	2.02 (1.11–3.67)
Prevalence of heart attack					
Model 1	1.00	1.31 (1.14–1.49)	2.73 (1.68–4.43)	1.86 (1.60–2.17)	2.95 (2.14–4.05)
Model 2	1.00	1.34 (1.10–1.62)	3.08 (1.59–5.95)	1.82 (1.45–2.27)	2.85 (1.85–4.38)
Model 3	1.00	1.32 (1.09–1.60)	3.22 (1.62–6.40)	1.59 (1.26–2.00)	2.26 (1.46–3.49)
Prevalence of stroke					
Model 1	1.00	1.11 (0.98–1.26)	1.59 (1.04–2.43)	1.52 (1.31–1.76)	1.81 (1.31–2.51)
Model 2	1.00	1.07 (0.89–1.29)	1.57 (0.94–2.63)	1.45 (1.18–1.79)	1.67 (1.10–2.52)
Model 3	1.00	1.02 (0.84–1.23)	1.62 (0.97–2.71)	1.23 (0.99–1.52)	1.26 (0.83–1.90)
From 10 years before baseline to baseline					
Prevalence of cardiovascular diseases					
Model 1	1.00	1.36 (1.22–1.51)	2.22 (1.90–2.59)	2.15 (1.88–2.46)	2.32 (2.04–2.64)
Model 2	1.00	1.32 (1.14–1.54)	2.16 (1.75–2.67)	2.06 (1.69–2.51)	2.28 (1.91–2.73)
Model 3	1.00	1.27 (1.09–1.49)	2.00 (1.61–2.48)	1.74 (1.41–2.16)	1.88 (1.56–2.26)
Prevalence of congestive heart failure					
Model 1	1.00	1.30 (1.06–1.61)	2.64 (2.05–3.40)	2.51 (1.98–3.20)	3.23 (2.58–4.05)
Model 2	1.00	1.24 (0.91–1.68)	2.67 (1.84–3.86)	2.49 (1.76–3.52)	3.38 (2.52–4.53)
Model 3	1.00	1.18 (0.87–1.60)	2.59 (1.75–3.86)	2.15 (1.48–3.14)	2.83 (2.12–3.79)
Prevalence of coronary heart disease					
Model 1	1.00	1.36 (1.16–1.59)	1.89 (1.49–2.39)	2.04 (1.61–2.58)	2.17 (1.78–2.66)
Model 2	1.00	1.20 (0.97–1.50)	1.72 (1.25–2.36)	1.95 (1.37–2.77)	1.84 (1.36–2.50)
Model 3	1.00	1.12 (0.89–1.40)	1.56 (1.13–2.14)	1.62 (1.12–2.35)	1.52 (1.12–2.07)
Prevalence of angina pectoris					
Model 1	1.00	1.36 (1.11–1.64)	1.56 (1.20–2.04)	2.38 (1.90–2.97)	2.39 (1.92–2.99)
Model 2	1.00	1.29 (0.98–1.70)	1.50 (1.03–2.19)	1.96 (1.36–2.84)	2.19 (1.59–3.02)
Model 3	1.00	1.17 (0.88–1.56)	1.28 (0.88–1.87)	1.55 (1.05–2.30)	1.70 (1.23–2.36)
Prevalence of stroke					
Model 1	1.00	1.25 (1.07–1.47)	2.14 (1.71–2.67)	1.65 (1.34–2.04)	1.89 (1.58–2.27)
Model 2	1.00	1.15 (0.92–1.44)	1.88 (1.40–2.53)	1.49 (1.12–1.98)	1.76 (1.36–2.28)
Model 3	1.00	1.07 (0.85–1.35)	1.70 (1.25–2.31)	1.25 (0.94–1.65)	1.42 (1.09–1.86)

95% CI, 95% Confidence Interval; CVD, Cardiovascular diseases.

a All the estimated values have taken into account the complex sampling designs. Among 35,998 participants, a total of 1,512, 964, and 1,001 participants were excluded in analyses from age 25 years to 10 years before baseline, age 25 years to baseline, and 10 years before baseline to baseline, respectively, owing to missing values of body mass index at both times. Model 1 adjusted for baseline age, gender and race/ethnicity. Model 2 further adjusted for educational level, income level, marital status, alcohol consumption, smoking status, family history of diabetes, and family history of heart disease. Model 3 further adjusted for baseline moderate exercise status and general health condition.

When weight change patterns were evaluated across specific adulthood intervals, stable obesity and transition from non-obesity to obesity from young to middle adulthood were consistently associated with higher odds of prevalent CVD, with the ORs for CVD were 1.92 (1.38 to 2.65) and 1.66 (1.42 to 1.94) respectively. In contrast, transition from obesity to non-obesity from middle to older adulthood was associated with higher odds of prevalent CVD (OR, 2.00; 95% CI, 1.61–2.48). Analogous trends were identified for prevalent CVD in the context of absolute weight change trajectories (Supplementary Table S4).

### 3.3 Weight change patterns and major cardiovascular disease subtypes

Associations between weight change patterns and major CVD subtypes are summarized in Table 2. Stable obesity from young to middle adulthood was strongly associated with congestive heart failure, with a multivariable-adjusted OR of 3.49 (95% CI, 2.41–5.06). Transition from a non-obese to an obese state was consistently associated with an elevated risk of each of the five CVD subtypes.

From mid- to late adulthood, transition from an obese to a non-obese state was associated with an elevated risk of four CVD subtypes (excluding angina pectoris). Associations with stroke were generally weaker yet exhibited analogous directional trends. No consistent associations were detected for maximum overweight status across most CVD subtypes following multivariable adjustment.

### 3.4 Weight change patterns and cardiovascular disease mortality

During a median follow-up of 9.2 years (310,647 person-years), a total of 2,314 CVD-related deaths were documented. Associations between weight change patterns and CVD mortality are summarized in Table 3. Participants with stable obesity exhibited an elevated risk of CVD mortality from young to middle adulthood (HR, 1.84; 95% CI, 1.32–2.55). Relative to participants maintaining stable normal weight, persistent obesity across adulthood was associated with a higher risk of CVD mortality (HR, 1.55; 95% CI, 1.07–2.25). Weight transition from the obese to non-obese range across adulthood was associated with a 107% increased risk of CVD mortality (HR, 2.07; 95% CI, 1.25–3.43). Similarly, weight transition from obesity to non-obesity during mid- to late adulthood was also linked to an elevated risk of CVD mortality (HR, 1.58; 95% CI, 1.22–2.05).

**Table 3:** Hazard ratios (95% CIs) of CVD mortality with weight change patterns.^a^

	Weight change patterns				
	Stable normal	Maximum overweight	Obese to non-obese	Non-obese to obese	Stable obese
From age 25 years to 10 years before baseline					
No of deaths/person years	883/137951.92	835/114870.83	24/1852.00	482/59021.67	90/8832.67
Age adjusted mortality rate^b^	7.89 (7.05–8.72)	8.05 (7.17–8.94)	10.97 (3.80–18.13)	9.34 (7.90–10.78)	10.05 (6.52–13.58)
Model 1	1.00	0.98 (0.89–1.08)	1.65 (1.11–2.47)	1.33 (1.19–1.49)	1.33 (1.38–2.13)
Model 2	1.00	0.95 (0.81–1.12)	1.47 (0.73–2.98)	1.18 (0.99–1.42)	1.80 (1.30–2.50)
Model 3	1.00	0.94 (0.80–1.11)	1.34 (0.64–2.81)	1.15 (0.96–1.38)	1.84 (1.32–2.55)
From age 25 years to baseline					
No of deaths/person years	765/97725.33	881/119867.67	46/2726.75	554/94251.42	68/7957.92
Age adjusted mortality rate^b^	8.31 (7.36–9.27)	8.15 (7.26–9.04)	13.80 (7.28–20.40)	8.03 (6.92–9.14)	8.40 (4.89–11.91)
Model 1	1.00	0.98 (0.89–1.08)	1.80 (1.29–2.49)	1.14 (1.02–1.27)	1.54 (1.18–1.99)
Model 2	1.00	1.03 (0.88–1.22)	2.14 (1.31–3.51)	1.02 (0.85–1.23)	1.52 (1.05–2.21)
Model 3	1.00	1.01 (0.86–1.20)	2.07 (1.25–3.43)	1.00 (0.83–1.20)	1.55 (1.07–2.25)
From 10 years before baseline to baseline					
No of deaths/person years	661/87999.50	829/117632.08	202/14688.17	252/49043.17	370/53166.17
Age adjusted mortality rate^b^	8.14 (7.13–9.15)	7.81 (6.94–8.67)	13.24 (9.95–16.54)	8.23 (6.61–9.85)	8.27 (6.79–9.75)
Model 1	1.00	0.95 (0.86–1.05)	1.70 (1.45–2.00)	1.14 (0.98–1.32)	1.24 (1.09–1.41)
Model 2	1.00	0.99 (0.83–1.19)	1.63 (1.27–2.10)	1.00 (0.78–1.29)	1.14 (0.93–1.41)
Model 3	1.00	0.98 (0.81–1.17)	1.58 (1.22–2.05)	0.99 (0.77–1.27)	1.12 (0.90–1.38)

95% CI, 95% Confidence Interval; CVD, Cardiovascular diseases.

a All the estimated values have taken into account the complex sampling designs. Among 35,998 participants, a total of 1,512, 964, and 1,001 participants were excluded in analyses from age 25 years to 10 years before baseline, age 25 years to baseline, and 10 years before baseline to baseline, respectively, owing to missing values of body mass index at both times. Model 1 adjusted for baseline age, gender and race/ethnicity. Model 2 further adjusted for educational level, income level, marital status, alcohol consumption, smoking status, family history of diabetes, and family history of heart disease. Model 3 further adjusted for baseline moderate exercise status and general health condition.

b Mortality rates per 1,000 person years, directly standardised to age distribution of entire study population.

In contrast, transition from a non-obese to an obese state was not significantly associated with CVD mortality following multivariable adjustment. Associations between absolute weight change patterns and CVD mortality are summarized in Supplementary Table S6. Relative to participants maintaining stable weight (weight change within 2.5 kg), those in the extreme weight gain group (weight gain ≥20 kg) exhibited an elevated risk of CVD mortality during mid- to late adulthood (HR, 1.47; 95% CI, 1.07–2.04). Analogous findings were observed for participants with weight loss exceeding 2.5 kg both across adulthood and during mid- to late adulthood, with corresponding hazard ratios of 1.32 (95% CI, 1.03–1.67) and 1.40 (95% CI, 1.19–1.65), respectively. However, participants with weight gain ≥10 kg and <20 kg had a reduced risk of CVD mortality from young to mid-adulthood and across adulthood, with hazard ratios of 0.80 (95% CI, 0.66–0.98) and 0.72 (95% CI, 0.58–0.89), respectively. An inverse association with CVD mortality during mid- to late adulthood was also identified among participants with weight gain ≥2.5 kg and <10 kg (HR, 0.78; 95% CI, 0.63–0.96).

### 3.5 Subgroup analyses by age and sex

Findings of subgroup analyses stratified by age and sex are presented in Supplementary Tables S3, S5, S7, S8. Overall, associations between adverse weight change trajectories and prevalent CVD and CVD mortality tended to be stronger among participants aged ≤65 years than among those aged >65 years. In participants aged >65 years, the corresponding associations were generally attenuated. Sex-stratified results were broadly similar. Small-to-moderate weight gain showed inverse associations with CVD mortality in some sex-specific analyses, particularly among women. However, formal interaction tests were not statistically significant for most comparisons (most P for interaction > 0.05).

## 4 Discussion

### 4.1 Overview of key study findings

In this population-based study, we found that long-term weight change patterns across adulthood were differentially associated with CVD prevalence and mortality in a life-stage-specific manner. Persistent obesity and transition from non-obesity to obesity from young to middle adulthood were consistently associated with higher prevalent CVD and its major subtypes. In contrast, weight loss from middle to older adulthood, reflected by transition from obesity to non-obesity, was associated with higher risk of CVD prevalence and mortality. These associations were generally more pronounced among individuals younger than 65 years, highlighting potential heterogeneity by age. Gender differences are also observed in the association between different weight change patterns and CVD prevalence and mortality. Interestingly, we also found that small-to-moderate weight gain was inversely associated with CVD mortality in some analyses.

Combined with the research hypotheses proposed in the introduction, our study results fully confirmed hypothesis (1) and partially verified hypothesis (2). Specifically, sustained obesity and weight gain from early to middle adulthood significantly increased CVD prevalence and mortality. In terms of subgroup differences, the associations between adverse weight trajectories and CVD outcomes were stronger in adults aged ≤65 years than in those over 65 years, which supported the age-related part of Hypothesis (2); while most gender interaction tests showed no statistical significance, the gender-related part of Hypothesis (2) was not fully validated.

### 4.2 Comparison and linkage with previous relevant studies

Our findings align with and extend previous evidence linking adult long-term obesity and weight gain to adverse cardiovascular outcomes (16, 23–25). Several large prospective cohort studies have demonstrated that weight gain from early adulthood is associated with increased risks of coronary heart disease, heart failure, and stroke later in life (7, 21, 22, 26). Notably, studies comparing different life-course weight trajectories have suggested that sustained obesity beginning in early adulthood confers a higher cardiovascular risk than obesity developing later in life, supporting the concept of cumulative exposure to excess adiposity as a key determinant of cardiovascular damage (27).

Our results are also consistent with prior research emphasizing the particularly strong association between obesity and heart failure. Previous studies have shown that obesity is more strongly related to heart failure than to atherosclerotic outcomes, possibly due to obesity-related myocardial remodeling, increased plasma volume, and long-term hemodynamic overload (28). The elevated odds of congestive heart failure observed among individuals with stable obesity or early adult weight gain in our study are in line with these observations and reinforce the notion that prolonged exposure to excess body weight may be especially detrimental to myocardial structure and function (29).

Our finding that a certain degree of weight gain may confer a protective effect against CVD mortality is consistent with previous studies (30–32). One possible explanation is that the estrogen converted from fat tissue can provide protection for postmenopausal women with weight gain (30). Additionally, patients with lower body weight due to older age or concurrent with malignant diseases, malnutrition, or multi-organ dysfunction have a poorer prognosis for CVD (33). Furthermore, excess fat is more effective at providing energy and preventing the atrophy of lean tissue than exogenous nutrients, thereby reducing the mortality rate of CVD (33).

In contrast, evidence regarding the cardiovascular implications of weight loss has been heterogeneous. While randomized controlled trials and lifestyle intervention studies have demonstrated improvements in cardiometabolic risk factors following intentional weight reduction (34), observational studies-particularly among middle-aged and older adults-have frequently reported higher risks of mortality or cardiovascular events associated with weight loss (14). Our findings extend this literature by showing that weight loss occurring from middle to older adulthood, operationalized as transition from obesity to non-obesity, was associated with both higher CVD prevalence and increased CVD mortality at the population level. These results are consistent with prior reports suggesting that the prognostic meaning of weight loss may differ substantially according to age, health status, and underlying causes of weight change (5).

Importantly, few prior studies have simultaneously examined weight change patterns across multiple adulthood stages related to both prevalent CVD and mortality within a single analytic framework. By jointly evaluating early-to-midlife and midlife-to-late-life weight transitions, our study helps reconcile inconsistencies in the existing literature and highlights the importance of incorporating life-stage-specific perspectives when interpreting associations between weight change and cardiovascular outcomes (35).

### 4.3 Potential biological and pathophysiological mechanisms

Several biological and pathophysiological mechanisms may underlie the observed life-stage-specific associations between weight change patterns and cardiovascular outcomes. Prolonged exposure to excess adiposity beginning in early adulthood may lead to cumulative cardiometabolic stress through insulin resistance, dyslipidemia, chronic low-grade inflammation, oxidative stress, and endothelial dysfunction (36). Over time, these processes can promote atherosclerosis, myocardial remodeling, and impaired vascular function, thereby increasing susceptibility to coronary heart disease, heart failure, and stroke (37, 38).

From a cardiac-specific perspective, long-term obesity has been linked to increased blood volume, elevated cardiac output, and sustained hemodynamic loading, which may contribute to left ventricular hypertrophy, diastolic dysfunction, and eventual heart failure (39). These alterations may partially explain the particularly strong associations observed for congestive heart failure in our study. Importantly, such structural and functional changes may become less reversible with advancing age, underscoring the significance of weight trajectories earlier in the adult life course (40).

In contrast, weight loss occurring later in adulthood may reflect fundamentally different biological processes. In older populations, weight loss is often unintentional and may be driven by underlying chronic disease, subclinical cardiovascular pathology, systemic inflammation, or age-related decline in appetite and physical function (41). In addition, age-related changes in body composition-including preferential loss of skeletal muscle mass, sarcopenia, and reduced physiological reserve-may amplify vulnerability to adverse outcomes when weight loss occurs (42). Loss of lean mass rather than fat mass may be particularly detrimental for cardiovascular resilience and overall survival in later life (43).

### 4.4 Academic value of the findings and advancements to current knowledge

Taken together, these findings support a life-course framework in which the direction and timing of weight change carry distinct prognostic meanings. Weight gain and sustained obesity earlier in adulthood may contribute to the accumulation of irreversible cardiovascular damage, whereas weight loss later in life may serve as a marker of underlying vulnerability rather than a protective process (44). This perspective may help reconcile the apparent paradoxes observed in the literature and emphasizes the need to interpret weight change in the context of age, timing, and underlying health status when assessing cardiovascular risk (24).

### 4.5 Strengths of the study

This study has several notable strengths. We leveraged a representative sample of adults with long-term follow-up and standardized ascertainment of both prevalent CVD and CVD-related mortality. Weight change patterns were characterized across multiple adulthood intervals, allowing evaluation of life-stage-specific associations that are difficult to capture in studies relying on single time-point measurements (35). In addition, the use of survey-weighted analyses enhances the generalizability of our findings to groups of people in China.

### 4.6 Limitations of the study and their impact on result interpretation

Several limitations should also be acknowledged. Body weight at earlier adulthood was self-reported and may be subject to recall bias. Residual confounding and reverse causation cannot be fully excluded, particularly for analyses involving weight loss in later adulthood (45, 46). The inability to distinguish intentional from unintentional weight loss is an important limitation, as these processes may have divergent cardiovascular implications (44). Furthermore, prevalent CVD was based on self-reported physician diagnoses, which may lead to misclassification. Finally, observational design precludes causal inference.

## 5 Conclusion

In conclusion, long-term weight change patterns across adulthood were associated with cardiovascular disease risk and mortality in a life-stage-dependent manner in this representative population. Persistent obesity and weight gain beginning in early adulthood were associated with higher CVD risk, whereas weight loss occurring later in adulthood was associated with increased CVD mortality. These findings highlight the importance of considering the timing of weight change across the adult life course when evaluating cardiovascular risk.

## Data Availability

The data analyzed in this study is subject to the following licenses/restrictions: the dataset is not publicly available because it contains confidential participant health data protected by institutional ethics and privacy policies. Access to the data is restricted to authorized study investigators. Reasonable data access requests may be directed to the corresponding author. Requests to access these datasets should be directed to liju532@126.com.
