# Supplementary material for: Adult weight change across the life course and cardiovascular disease prevalence and mortality: a population-based study
Source: Front Public Health. 2026 Jul 2;14:1879396. doi: 10.3389/fpubh.2026.1879396 (PMC13372993; doi:10.3389/fpubh.2026.1879396)
Supplement: Supplementary file 1 [file Table_1.doc]

**Table S1 | Baseline characteristics of study population according to weight change patterns from age 25 years to 10 years before baseline.**

| **Characteristics†** | **Total** | **Weight change patterns from age 25 years to 10 years before baseline** | | | | |  |
| --- | --- | --- | --- | --- | --- | --- | --- |
|  |  | **Stable normal** | **Maximum**  **overweight** | **Obese to**  **non-obese** | **Non-obese to**  **obese** | **Stable obese** | ***P* value‡** |
| **Study population (n/%)** | 35998 | 14088 (39.14) | 12653 (35.15) | 227 (0.63) | 7904 (21.96) | 1126 (3.13) | - |
| **Age means (95% CI), years** | 57.53 (57.27-57.78) | 56.37 (56.06-56.68) | 58.34 (58.02-58.66) | 61.61 (59.97-63.25) | 57.95 (57.53-58.38) | 59.93 (59.37-60.49) | <0.01 |
| **Sex** |  |  |  |  |  |  |  |
| Male | 17541 (47.17) | 5665 (35.70) | 7501 (59.41) | 124 (51.27) | 3669 (47.79) | 582 (52.65) | <0.01 |
| Female | 18457 (52.83) | 8423 (63.30) | 5152 (40.59) | 103 (48.73) | 4235 (52.21) | 544 (47.35) |  |
| **BMI means (95% CI), kg/m^2^** | |  |  |  |  |  |  |
| At the age of 25 | 21.03 (20.94-21.11) | 20.36 (20.24-20.41) | 22.52 (22.46-22.59) | 31.74 (31.55-32.38) | 22.85 (22.74-23.26) | 28.36 (28.05-28.67) | <0.01 |
| At 10 years prior to baseline | 25.04 (24.93-25.16) | 21.31 (21.25-21.40) | 25.67 (25.61-25.72) | 24.47 (24.02-25.31) | 26.34 (26.20-26.46) | 32.84 (32.37-33.31) | <0.01 |
| At baseline survey | 27.39 (27.27-27.51) | 22.30 (22.26-22.48) | 26.20 (26.16-26.34) | 26.64 (26.02-27.12) | 29.92 (29.83-30.11) | 30.23 (30.02-30.76) | <0.01 |
| Absolute weight change mean (95% CI), kg | 11.84 (11.59-12.09) | 2.52 (2.33-2.75) | 7.20 (7.12-7.29) | -14.69 (-15.70--13.58) | 18.44 (18.02-18.79) | 9.17 (7.53-11.71) | <0.01 |
| **Race or ethnicity (n/%)** | |  |  |  |  |  |  |
| Han | 35711(99.20) | 14957 (99.52) | 10993 (99.36) | 292 (97.41) | 8628 (99.01) | 841 (97.79) | <0.01 |
| Others | 287 (0.80) | 173 (0.48) | 71 (0.64) | 8(0.59) | 16 (0.99) | 19 (2.21) |  |
| **Marital status (n/%)** |  |  |  |  |  |  |  |
| Married or living with partner | 21869 (67.36) | 8805 (66.17) | 8140 (70.23) | 127 (59.07) | 4185 (65.58) | 612 (63.10) | <0.01 |
| Others | 13751 (32.64) | 5865 (33.84) | 4433 (29.77) | 107 (40.93) | 2853 (34.42) | 493 (36.90) |  |
| **Annual family income (n/%)** | |  |  |  |  |  |  |
| Less than ¥9999 | 12334 (24.27) | 4878 (45.66) | 4930 (39.47) | 224 (63.46) | 1904 (21.43) | 398 (25.35) | <0.01 |
| ¥10000 to ¥19999 | 14296 (42.93) | 3404(31.86) | 5000 (40.03) | 105 (29.75) | 4881 (54.93) | 906 (57.71) |  |
| ¥20000 and over | 7353 (32.80) | 2401 (22.48) | 2561 (20.50) | 24 (6.79) | 2101 (23.64) | 266 (16.94) |  |
| **Educational levels (n/%)** | |  |  |  |  |  |  |
| Below high school | 34366 (95.72) | 10499 (95.21) | 11872 (95.93) | 415 (96.74) | 10208 (96.20) | 1372 (95.01) | <0.01 |
| High school or above | 1537 (4.28) | 528 (4.79) | 504 (4.07) | 14 (3.26) | 403 (3.80) | 72 (4.99) |  |
| **Alcohol drinking (n/%)** | |  |  |  |  |  |  |
| Yes | 20210 (75.93) | 7829 (75.03) | 7517 (78.52) | 155 (73.65) | 4075 (73.80) | 634 (72.76) | <0.01 |
| No | 8460 (24.07) | 3591 (24.97) | 2676 (21.48) | 49 (26.35) | 1849 (26.20) | 295 (27.24) |  |
| **Smoking status (n/%)** | |  |  |  |  |  |  |

**Continuous Table S1 | Baseline characteristics of study population according to weight change patterns from age 25 years to 10 years before baseline.**

| **Characteristics†** | **Total** | **Weight change patterns from age 25 years to 10 years before baseline** | | | | | ***P* value‡** |
| --- | --- | --- | --- | --- | --- | --- | --- |
|  |  | **Stable normal** | **Maximum**  **overweight** | **Obese to**  **non-obese** | **Non-obese to**  **obese** | **Stable obese** |  |
| Yes | 17738 (49.35) | 5303 (48.50) | 6652 (49.77) | 215 (59.00) | 5069 (49.49) | 499 (49.26) | 0.13 |
| No | 18212 (50.65) | 5753 (51.50) | 6488 (50.23) | 158 (41.00) | 5333 (50.51) | 480 (50.74) |  |
| **Moderate activity (n/%)** | |  |  |  |  |  |  |
| Yes | 12889 (43.20) | 5139 (42.87) | 4769 (44.74) | 71 (36.76) | 2532 (41.95) | 378 (38.56) | <0.01 |
| No | 22279 (56.80) | 9380 (57.13) | 7642 (55.26) | 156 (63.24) | 4396 (58.05) | 705 (61.44) |  |
| **General health condition (n/%)** | |  |  |  |  |  |  |
| Very good to excellent | 12756 (44.96) | 5944 (50.58) | 4654 (45.57) | 65 (36.55) | 1854 (34.71) | 239 (27.70) | <0.01 |
| Good | 12666 (33.96) | 4989 (30.60) | 4588 (35.35) | 78 (30.36) | 2597 (37.73) | 414 (40.52) |  |
| Poor to fair | 10550 (21.08) | 3914 (18.82) | 3450 (19.08) | 93 (33.09) | 2630 (27.56) | 463 (31.78) |  |
| **Family history of heart attack (n/%)** | |  |  |  |  |  |  |
| Yes | 4227 (18.08) | 1571 (17.41) | 1403 (16.54) | 34 (22.65) | 1048 (21.54) | 171 (19.59) | <0.01 |
| No | 21909 (81.92) | 8880 (82.59) | 7650 (83.46) | 141 (77.35) | 4531 (78.46) | 707 (80.41) |  |
| **Family history of diabetes (n/%)** | |  |  |  |  |  |  |
| Yes | 14543 (48.40) | 5112 (43.38) | 5107 (48.15) | 116 (55.17) | 3603 (56.62) | 605 (59.48) | <0.01 |
| No | 14238 (51.60) | 6365 (56.62) | 4953 (51.85) | 81 (44.83) | 2476 (43.38) | 363 (40.52) |  |
| **Cardiovascular diseases (n/%)** | |  |  |  |  |  |  |
| Yes | 6112 (13.35) | 1934 (9.83) | 2275 (14.19) | 59 (20.45) | 1546 (17.76) | 298 (22.36) | <0.01 |
| No | 29886 (86.65) | 12927 (90.17) | 10421 (85.81) | 177 (79.55) | 5543 (82.24) | 818 (77.64) |  |
| **Congestive heart failure (n/%)** | |  |  |  |  |  |  |
| Yes | 1852 (3.71) | 511 (2.43) | 632 (3.55) | 26 (8.08) | 559 (5.89) | 124 (8.59) | <0.01 |
| No | 34146 (96.29) | 14350 (97.58) | 12064 (96.45) | 210 (91.92) | 6530 (94.11) | 992 (91.41) |  |
| **Coronary heart disease (n/%)** | |  |  |  |  |  |  |
| Yes | 2310 (5.42) | 697 (3.76) | 910 (6.03) | 27 (9.98) | 560 (7.05) | 116 (10.21) | <0.01 |
| No | 33688 (94.58) | 14164 (96.24) | 11786 (93.97) | 209 (90.02) | 6529 (92.95) | 1000 (89.79) |  |
| **Angina pectoris (n/%)** | |  |  |  |  |  |  |
| Yes | 1573 (3.76) | 452 (2.67) | 606 (4.08) | 14 (5.14) | 421 (5.00) | 80 (6.99) | <0.01 |
| No | 34425 (96.24) | 14409 (97.33) | 12090 (95.92) | 222 (94.86) | 6668 (95.00) | 1036 (93.01) |  |
| **Heart attack (n/%)** |  |  |  |  |  |  |  |

**Continuous Table S1 | Baseline characteristics of study population according to weight change patterns from age 25 years to 10 years before baseline.**

| **Characteristics†** | **Total** | **Weight change patterns from age 25 years to 10 years before baseline** | | | | | ***P* value‡** |
| --- | --- | --- | --- | --- | --- | --- | --- |
|  |  | **Stable normal** | **Maximum**  **overweight** | **Obese to**  **non-obese** | **Non-obese to**  **obese** | **Stable obese** |  |
| Yes | | 2397 (5.30) | 707 (3.64) | 915 (5.78) | 27 (8.19) | 608 (7.03) | <0.01 |
| No | | 33601 (94.70) | 14154 (96.36) | 11781 (94.22) | 209 (91.81) | 6481 (92.97) |  |
| **Stroke (n/%)** | |  |  |  |  |  |  |
| Yes | | 2117 (4.30) | 710 (3.42) | 776 (4.29) | 17 (7.03) | 516 (5.84) | <0.01 |
| No | | 33881 (95.70) | 14151 (96.58) | 11920 (95.71) | 219 (92.97) | 6573 (94.16) |  |

Abbreviations: 95% CI, 95% Confidence Interval; BMI, Body Mass Index.

*All estimates accounted for complex survey designs.

**†**Regarding the information on baseline educational level, marital status, family annual income, drinking habits, overall health condition, moderate exercise status, smoking habits, family history of heart attack and Family history of diabetes, there were 95, 378, 2015 , 7328 , 26, 830, 48, 9862 and 7217 participants who had missing information respectively.

‡ The *P* values for categorical variables are calculated using the Rao-Scott chi-square test, which is a modified version of the Pearson chi-square test. The *P* values for continuous variables are calculated through variance analysis that takes into account the adjustment of sampling weights.

**Table S2 | Baseline characteristics of study population according to weight change patterns from age 10 years before baseline to baseline.**

| **Characteristics†** | **Total** | **Weight change patterns from age 10 years before baseline to baseline** | | | | |  |
| --- | --- | --- | --- | --- | --- | --- | --- |
|  |  | **Stable normal** | **Maximum**  **overweight** | **Obese to**  **non-obese** | **Non-obese to**  **obese** | **Stable obese** | ***P* value‡** |
| **Study population (n/%)** | 35998 | 9832 (27.31) | 12815 (35.60) | 1966 (5.46) | 5146 (14.30) | 6239 (17.33) | - |
| **Age means (95% CI), years** | 57.53 (57.27-57.78) | 58.21 (57.82-58.60) | 57.93 (57.59-58.26) | 62.28 (61.07-63.50) | 56.00 (55.64-56.37) | 59.59 (59.01-60.16) | ＜0.01 |
| **Sex** |  |  |  |  |  |  |  |
| Male | 17541 (47.17) | 4071 (35.70) | 7376 (56.35) | 1034 (51.30) | 2152 (42.87) | 2908 (48.79) | ＜0.01 |
| Female | 18457 (52.83) | 5761 (64.30) | 5439 (43.65) | 932 (48.70) | 2994 (57.13) | 3331 (51.21) |  |
| **BMI means (95% CI), kg/m^2^** |  |  |  |  |  |  |  |
| At the age of 25 | 21.62 (21.24-21.97) | 20.25 (20.11-20.37) | 21.45 (21.38-21.50) | 32.65 (32.52-32.74) | 23.46 (24.35-24.60) | 29.36 (29.03-29.64) | ＜0.01 |
| At 10 years prior-baseline | 24.55 (24.33-24.86) | 21.31 (21.22-21.44) | 25.39 (25.29-25.48) | 25.17 (25.01-25.29) | 26.19 (26.02-26.27) | 31.72 (31.55-31.90) | ＜0.01 |
| At baseline survey | 27.82 (27.62-27.99) | 23.30 (23.21-23.46) | 26.71 (26.63-26.81) | 27.73 (27.66-27.86) | 31.53 (31.40-31.71) | 30.23 (30.02-30.44) | ＜0.01 |
| Absolute weight change mean (95% CI), kg | 6.65 (6.51-6.72) | 3.36 (3.14-3.48) | 6.89 (6.77-6.96) | -15.45 (-16.13--14.21) | 15.44 (15.12-15.77) | 6.53 (6.41-6.62) | ＜0.01 |
| **Race or ethnicity (n/%)** |  |  |  |  |  |  |  |
| Han | 35711(99.20) | 14970 (98.94) | 10970 (99.15) | 285 (95.00) | 8630 (99.84) | 856 (99.53) | ＜0.01 |
| Others | 287 (0.80) | 160 (1.06) | 94 (0.85) | 15(5.00) | 14 (0.16) | 4 (0.47) |  |
| **Marital status (n/%)** |  |  |  |  |  |  |  |
| Married or living with partner | 21869 (67.36) | 5796 (65.97) | 8224 (70.49) | 1101 (63.61) | 3052 (65.51) | 3696 (65.72) | ＜0.01 |
| Others | 13751 (32.64) | 3905 (34.03) | 4463 (29.51) | 852 (36.39) | 2037 (34.49) | 2494 (34.28) |  |
| **Annual family income (n/%)** |  |  |  |  |  |  |  |
| Less than ¥9999 | 12334 (24.27) | 4931 (46.51) | 4785 (38.52) | 354 (71.08) | 1827 (21.59) | 437 (21.87) | ＜0.01 |
| ¥10000 to ¥19999 | 14296 (42.93) | 3351(31.61) | 5214 (41.97) | 101 (20.28) | 4623 (54.62) | 1007 (50.40) |  |
| ¥20000 and over | 7353 (32.80) | 2319 (21.88) | 2423 (19.51) | 43 (8.64) | 2014 (23.79) | 554 (27.73) |  |
| **Educational levels (n/%)** |  |  |  |  |  |  |  |
| Below high school | 34366 (95.72) | 10258 (96.10) | 11623 (95.18) | 550 (93.70) | 10005 (95.91) | 1930 (96.60) | ＜0.01 |
| High school or above | 1537 (4.28) | 416 (3.90) | 589 (4.82) | 37 (6.30) | 427 (4.09) | 68 (3.40) |  |
| **Alcohol drinking (n/%)** |  |  |  |  |  |  |  |
| Yes | 20210 (75.93) | 5025 (75.23) | 7564 (78.73) | 1128 (76.57) | 2912 (74.06) | 3581 (72.88) | ＜0.01 |
| No | 8460 (24.07) | 2386 (24.77) | 2641 (21.27) | 463 (23.43) | 1289 (25.94) | 1681 (27.12) |  |
| **Smoking status (n/%)** |  |  |  |  |  |  |  |
| Yes | 17738 (49.35) | 4652 (48.05) | 6505 (50.01) | 1014 (51.02) | 2605 (51.82) | 2962 (47.59) | ＜0.01 |
| No | 18212 (50.65) | 5148 (51.95) | 6300 (49.99) | 952 (48.98) | 2537 (48.18) | 3275 (52.41) |  |
| **Moderate activity (n/%)** |  |  |  |  |  |  |  |
| Yes | 12889 (43.20) | 3348 (42.73) | 4770 (44.84) | 644 (41.44) | 1861 (42.61) | 2266 (41.54) | 0.0186 |
| No | 22279 (56.80) | 6215 (57.27) | 7791 (55.16) | 1260 (58.56) | 3172 (57.39) | 3841 (58.46) |  |
| **General health condition (n/%)** | |  |  |  |  |  |  |
| Very good to excellent | 12756 (44.96) | 4181 (54.03) | 5072 (49.78) | 545 (37.97) | 1410 (33.03) | 1548 (32.66) | ＜0.01 |
| Good | 12666 (33.96) | 3154 (28.59) | 4518 (32.68) | 696 (35.71) | 1983 (41.02) | 2315 (38.75) |  |
| Poor to fair | 10550 (21.08) | 2489 (17.38) | 3220 (17.54) | 722 (26.32) | 1748 (25.95) | 2371 (28.59) |  |
| **Family history of heart attack (n/%)** | |  |  |  |  |  |  |
| Yes | 4227 (18.08) | 954 (16.10) | 1371 (16.62) | 280 (23.13) | 683 (19.95) | 939 (20.78) | ＜0.01 |
| No | 21909 (81.92) | 5957 (83.90) | 7672 (83.38) | 1243 (76.87) | 3042 (80.05) | 3995 (79.22) |  |
| **Family history of diabetes (n/%)** | |  |  |  |  |  |  |
| Yes | 14543 (48.40) | 3077 (39.74) | 4858 (46.13) | 957 (54.96) | 2400 (55.69) | 3251 (57.55) | ＜0.01 |
| No | 14238 (51.60) | 4441 (60.26) | 5172 (53.88) | 700 (45.04) | 1786 (44.31) | 2139 (42.45) |  |
| **Cardiovascular diseases (n/%)** | |  |  |  |  |  |  |
| Yes | 6112 (13.35) | 1285 (9.55) | 2121 (12.77) | 535 (22.34) | 862 (14.21) | 1309 (17.24) | ＜0.01 |
| No | 29886 (86.65) | 8547 (90.45) | 10694 (87.23) | 1431 (77.66) | 4284 (85.79) | 4930 (82.76) |  |
| **Congestive heart failure (n/%)** | |  |  |  |  |  |  |
| Yes | 1852 (3.71) | 331 (2.30) | 573 (3.02) | 181 (7.00) | 265 (4.17) | 502 (6.02) | ＜0.01 |
| No | 34146 (96.29) | 9501 (97.70) | 12242 (96.98) | 1785 (93.00) | 4881 (95.83) | 5737 (93.98) |  |
| **Coronary heart disease (n/%)** |  |  |  |  |  |  |  |
| Yes | 2310 (5.42) | 473 (3.72) | 870 (5.49) | 193 (8.80) | 291 (5.33) | 483 (7.07) | ＜0.01 |
| No | 33688 (94.58) | 9359 (96.28) | 11945 (94.51) | 1773 (91.20) | 4855 (94.67) | 5756 (92.93) |  |
| **Angina pectoris (n/%)** |  |  |  |  |  |  |  |
| Yes | 1573 (3.76) | 298 (2.51) | 527 (3.49) | 134 (4.96) | 247 (4.49) | 367 (5.34) | ＜0.01 |
| No | 34425 (96.24) | 9534 (97.49) | 12288 (96.51) | 1832 (95.04) | 4899 (95.51) | 5872 (94.66) |  |
| **Heart attack (n/%)** |  |  |  |  |  |  |  |
| Yes | 2397 (5.30) | 489 (3.62) | 847 (5.10) | 225 (9.78) | 313 (5.46) | 523 (6.96) | ＜0.01 |
| No | 33601 (94.70) | 9343 (96.38) | 11968 (94.90) | 1741 (90.22) | 4833 (94.54) | 5716 (93.04) |  |
| **Stroke (n/%)** |  |  |  |  |  |  |  |
| Yes | 2117 (4.30) | 461 (3.33) | 742 (4.01) | 202 (8.03) | 300 (4.39) | 412 (5.30) | ＜0.01 |
| No | 33881 (95.70) | 9371 (96.67) | 12073 (95.99) | 1764 (91.97) | 4846 (95.61) | 5827 (94.70) |  |

**Continuous Table S2 | Baseline characteristics of study population according to weight change patterns from age 10 years before baseline to baseline.**

| **Characteristics†** | **Total** | **Weight change patterns from age 10 years before baseline to baseline** | | | | |  |
| --- | --- | --- | --- | --- | --- | --- | --- |
|  |  | **Stable normal** | **Maximum**  **overweight** | **Obese to**  **non-obese** | **Non-obese to**  **obese** | **Stable obese** | ***P* value‡** |
| Yes | 17738 (49.35) | 4652 (48.05) | 6505 (50.01) | 1014 (51.02) | 2605 (51.82) | 2962 (47.59) | ＜0.01 |
| No | 18212 (50.65) | 5148 (51.95) | 6300 (49.99) | 952 (48.98) | 2537 (48.18) | 3275 (52.41) |  |
| **Moderate activity (n/%)** |  |  |  |  |  |  |  |
| Yes | 12889 (43.20) | 3348 (42.73) | 4770 (44.84) | 644 (41.44) | 1861 (42.61) | 2266 (41.54) | 0.0186 |
| No | 22279 (56.80) | 6215 (57.27) | 7791 (55.16) | 1260 (58.56) | 3172 (57.39) | 3841 (58.46) |  |
| **General health condition (n/%)** | |  |  |  |  |  |  |
| Very good to excellent | 12756 (44.96) | 4181 (54.03) | 5072 (49.78) | 545 (37.97) | 1410 (33.03) | 1548 (32.66) | ＜0.01 |
| Good | 12666 (33.96) | 3154 (28.59) | 4518 (32.68) | 696 (35.71) | 1983 (41.02) | 2315 (38.75) |  |
| Poor to fair | 10550 (21.08) | 2489 (17.38) | 3220 (17.54) | 722 (26.32) | 1748 (25.95) | 2371 (28.59) |  |
| **Family history of heart attack (n/%)** | |  |  |  |  |  |  |
| Yes | 4227 (18.08) | 954 (16.10) | 1371 (16.62) | 280 (23.13) | 683 (19.95) | 939 (20.78) | ＜0.01 |
| No | 21909 (81.92) | 5957 (83.90) | 7672 (83.38) | 1243 (76.87) | 3042 (80.05) | 3995 (79.22) |  |
| **Family history of diabetes (n/%)** | |  |  |  |  |  |  |
| Yes | 14543 (48.40) | 3077 (39.74) | 4858 (46.13) | 957 (54.96) | 2400 (55.69) | 3251 (57.55) | ＜0.01 |
| No | 14238 (51.60) | 4441 (60.26) | 5172 (53.88) | 700 (45.04) | 1786 (44.31) | 2139 (42.45) |  |
| **Cardiovascular diseases (n/%)** | |  |  |  |  |  |  |
| Yes | 6112 (13.35) | 1285 (9.55) | 2121 (12.77) | 535 (22.34) | 862 (14.21) | 1309 (17.24) | ＜0.01 |
| No | 29886 (86.65) | 8547 (90.45) | 10694 (87.23) | 1431 (77.66) | 4284 (85.79) | 4930 (82.76) |  |
| **Congestive heart failure (n/%)** |  |  |  |  |  |  |  |
| Yes | 1852 (3.71) | 331 (2.30) | 573 (3.02) | 181 (7.00) | 265 (4.17) | 502 (6.02) | ＜0.01 |
| No | 34146 (96.29) | 9501 (97.70) | 12242 (96.98) | 1785 (93.00) | 4881 (95.83) | 5737 (93.98) |  |
| **Coronary heart disease (n/%)** |  |  |  |  |  |  |  |
| Yes | 2310 (5.42) | 473 (3.72) | 870 (5.49) | 193 (8.80) | 291 (5.33) | 483 (7.07) | ＜0.01 |
| No | 33688 (94.58) | 9359 (96.28) | 11945 (94.51) | 1773 (91.20) | 4855 (94.67) | 5756 (92.93) |  |
| **Angina pectoris (n/%)** |  |  |  |  |  |  |  |
| Yes | 1573 (3.76) | 298 (2.51) | 527 (3.49) | 134 (4.96) | 247 (4.49) | 367 (5.34) | ＜0.01 |
| No | 34425 (96.24) | 9534 (97.49) | 12288 (96.51) | 1832 (95.04) | 4899 (95.51) | 5872 (94.66) |  |
| **Heart attack (n/%)** |  |  |  |  |  |  |  |

**Continuous Table S2 | Baseline characteristics of study population according to weight change patterns from age 10 years before baseline to baseline.**

| **Characteristics†** | **Total** | **Weight change patterns from age 10 years before baseline to baseline** | | | | |  |
| --- | --- | --- | --- | --- | --- | --- | --- |
|  |  | **Stable normal** | **Maximum**  **overweight** | **Obese to**  **non-obese** | **Non-obese to**  **obese** | **Stable obese** | ***P* value‡** |
| **Moderate activity (n/%)** |  |  |  |  |  |  |  |
| Yes | 2397 (5.30) | 489 (3.62) | 847 (5.10) | 225 (9.78) | 313 (5.46) | 523 (6.96) | ＜0.01 |
| No | 33601 (94.70) | 9343 (96.38) | 11968 (94.90) | 1741 (90.22) | 4833 (94.54) | 5716 (93.04) |  |
| **Stroke (n/%)** |  |  |  |  |  |  |  |
| Yes | 2117 (4.30) | 461 (3.33) | 742 (4.01) | 202 (8.03) | 300 (4.39) | 412 (5.30) | ＜0.01 |
| No | 33881 (95.70) | 9371 (96.67) | 12073 (95.99) | 1764 (91.97) | 4846 (95.61) | 5827 (94.70) |  |

Abbreviations: 95% CI, 95% Confidence Interval; BMI, Body Mass Index.

*All estimates accounted for complex survey designs.

**†**Regarding the information on educational level, marital status, family annual income, drinking habits, overall health condition, moderate exercise status, smoking habits, family history of heart attack and Family history of diabetes, there were 95, 378, 2015 , 7328 , 26, 830, 48, 9862 and 7217 participants who had missing information respectively.

‡ The *P* values for categorical variables are calculated using the Rao-Scott chi-square test, which is a modified version of the pearson chi-square test. The *P* values for continuous variables are calculated through variance analysis that takes into account the adjustment of sampling weights.

**Table S3 | Odds ratio (95% CIs) of CVD with weight change patterns stratified by age and gender*.**

|  | **Weight change patterns** | | | | |
| --- | --- | --- | --- | --- | --- |
|  | **Stable normal** | **Maximum overweight** | **Obese to non-obese** | **Non-obese to obese** | **Stable obese** |
| **From age 25 years to 10 years before baseline** |  |  |  |  |  |
| **Prevalence of cardiovascular diseases** |  |  |  |  |  |
| Stratified by age , years |  |  |  |  |  |
| ≤65 | 1.00 | 1.23 (0.98-1.55) | 1.02 (0.48-2.17) | 1.75 (1.40-2.19) | 2.23 (1.48-3.36) |
| ＞65 | 1.00 | 1.23 (1.07-1.40) | 1.40 (0.66-3.00) | 1.55 (1.32-1.83) | 1.32 (0.91-1.91) |
| The *P* value of interaction between age group and Weight change patterns | - | 0.3990 | 0.6181 | 0.8249 | 0.0749 |
| Stratified by gender |  |  |  |  |  |
| Male | 1.00 | 1.19 (0.95-1.50) | 0.75 (0.34-1.65) | 1.68 (1.36-2.08) | 2.17 (1.36-3.44) |
| Female | 1.00 | 1.28 (1.06-1.54) | 1.75 (0.88-3.46) | 1.59 (1.27-1.99) | 1.51 (1.07-2.15) |
| The *P* value of interaction between gender group and Weight change patterns | - | 0.9201 | 0.0910 | 0.6788 | 0.2928 |
| **Prevalence of congestive heart failure** |  |  |  |  |  |
| Stratified by age , years |  |  |  |  |  |
| ≤65 | 1.00 | 0.97 (0.64-1.47) | 1.35 (0.54-3.35) | 1.93 (1.36-2.75) | 3.73 (2.27-6.11) |
| ＞65 | 1.00 | 1.36 (0.99-1.86) | 3.39 (1.38-8.31) | 2.17 (1.61-2.93) | 2.19 (1.37-3.48) |
| The *P* value of interaction between age group and Weight change patterns | - | 0.1595 | 0.2487 | 0.6848 | 0.0660 |
| Stratified by gender |  |  |  |  |  |
| Male | 1.00 | 1.21 (0.85-1.72) | 1.16 (0.50-2.73) | 2.02 (1.52-2.68) | 3.20 (1.76-5.83) |
| Female | 1.00 | 1.16 (0.79-1.70) | 4.15 (1.87-9.22) | 2.12 (1.42-3.16) | 3.23 (2.11-4.93) |
| The *P* value of interaction between gender group and Weight change patterns | - | 0.7945 | 0.0445 | 0.8850 | 0.9048 |
| **Prevalence of coronary heart disease** |  |  |  |  |  |
| Stratified by age , years |  |  |  |  |  |
| ≤65 | 1.00 | 1.07 (0.74-1.54) | 1.44 (0.51-4.07) | 1.45 (0.97-2.16) | 2.26 (1.24-4.13) |
| ＞65 | 1.00 | 1.08 (0.87-1.33) | 2.58 (0.98-6.79) | 1.17 (0.91-1.52) | 1.24 (0.70-1.20) |
| The *P* value of interaction between age group and Weight change patterns | - | 0.7700 | 0.4575 | 0.6470 | 0.1708 |
| Stratified by gender |  |  |  |  |  |

**Continuous Table S3 | Odds ratio (95% CIs) of CVD with weight change patterns stratified by age and gender*.**

|  | **Weight change patterns** | | | | |
| --- | --- | --- | --- | --- | --- |
|  | **Stable normal** | **Maximum overweight** | **Obese to non-obese** | **Non-obese to obese** | **Stable obese** |
| Male | 1.00 | 0.96 (0.72-1.27) | 1.31 (0.53-3.21) | 1.30 (0.98-1.74) | 2.16 (1.26-3.71) |
| Female | 1.00 | 1.41 (1.01-1.98) | 3.45 (1.28-9.34) | 1.25 (0.86-1.83) | 1.12 (0.45-2.82) |
| The *P* value of interaction between gender group and Weight change patterns | - | 0.1267 | 0.1202 | 0.8206 | 0.2720 |
| **Prevalence of angina pectoris** |  |  |  |  |  |
| Stratified by age , years |  |  |  |  |  |
| ≤65 | 1.00 | 0.99 (0.66-1.49) | 0.92 (0.25-3.40) | 1.28 (0.87-1.86) | 1.87 (0.93-3.75) |
| ＞65 | 1.00 | 1.26 (0.91-1.73) | 2.62 (0.83-8.28) | 1.50 (1.10-2.05) | 1.03 (0.52-2.06) |
| The *P* value of interaction between age group and Weight change patterns | - | 0.2586 | 0.2669 | 0.5523 | 0.1957 |
| Stratified by gender |  |  |  |  |  |
| Male | 1.00 | 0.95 (0.63-1.43) | 0.42 (0.14-1.28) | 1.37 (0.92-2.05) | 2.34 (1.18-4.61) |
| Female | 1.00 | 1.39 (0.96-2.01) | 3.42 (1.22-9.53) | 1.32 (0.90-1.95) | 0.88 (0.32-2.38) |
| The *P* value of interaction between gender group and Weight change patterns | - | 0.1723 | 0.0050 | 0.9864 | 0.1589 |
| **Prevalence of heart attack** |  |  |  |  |  |
| Stratified by age , years |  |  |  |  |  |
| ≤65 | 1.00 | 1.16 (0.80-1.67) | 1.27 (0.44-3.63) | 1.68 (1.21-2.32) | 3.05 (1.76-5.31) |
| ＞65 | 1.00 | 1.18 (0.93-1.50) | 0.98 (0.28-3.41) | 1.35 (1.01-1.80) | 1.30 (0.84- 2.02) |
| The *P* value of interaction between age group and Weight change patterns | - | 0.5818 | 0.7130 | 0.4396 | 0.0223 |
| Stratified by gender |  |  |  |  |  |
| Male | 1.00 | 1.24 (0.95-1.62) | 1.19 (0.42-3.33) | 1.84 (1.38-2.46) | 3.09 (1.77-5.40) |
| Female | 1.00 | 1.17 (0.84-1.62) | 1.13 (0.32-4.07) | 1.13 (0.78-1.64) | 1.55 (0.80-3.00) |
| The *P* value of interaction between gender group and Weight change patterns | - | 0.6113 | 0.9912 | 0.0422 | 0.1454 |
| **Prevalence of stroke** |  |  |  |  |  |
| Stratified by age , years |  |  |  |  |  |
| ≤65 | 1.00 | 1.04 (0.75-1.46) | 0.77 (0.24-2.54) | 1.66 (1.18-2.33) | 1.60 (0.94-2.70) |
| ＞65 | 1.00 | 1.20 (0.93-1.54) | 1.53 (0.49-4.76) | 1.37 (1.08-1.74) | 1.29 (0.81-2.05) |

**Continuous Table S3 | Odds ratio (95% CIs) of CVD with weight change patterns stratified by age and gender*.**

|  | **Weight change patterns** | | | | |
| --- | --- | --- | --- | --- | --- |
|  | **Stable normal** | **Maximum overweight** | **Obese to non-obese** | **Non-obese to obese** | **Stable obese** |
| The *P* value of interaction between age group and Weight change patterns | - | 0.3289 | 0.4741 | 0.4061 | 0.5284 |
| Stratified by gender |  |  |  |  |  |
| Male | 1.00 | 1.24 (0.91-1.68) | 0.41 (0.09-1.78) | 1.65 (1.21-2.24) | 2.05 (1.07-3.92) |
| Female | 1.00 | 1.08 (0.85-1.37) | 1.83 (0.68-4.93) | 1.40 (1.04-1.88) | 1.18 (0.77-1.82) |
| The *P* value of interaction between gender group and Weight change patterns | - | 0.4929 | 0.1334 | 0.4711 | 0.2156 |
| **From age 25 years to baseline** |  |  |  |  |  |
| **Prevalence of cardiovascular diseases** |  |  |  |  |  |
| Stratified by age , years |  |  |  |  |  |
| ≤65 | 1.00 | 1.30 (1.03-1.66) | 2.72 (1.33-5.55) | 1.90 (1.47-2.47) | 2.26 (1.50-3.40) |
| ＞65 | 1.00 | 1.29 (1.12-1.50) | 1.29 (0.74-2.25) | 1.51 (1.24-1.83) | 1.41 (0.90-2.19) |
| The *P* value of interaction between age group and Weight change patterns | - | 0.5395 | 0.1006 | 0.2541 | 0.1416 |
| Stratified by gender |  |  |  |  |  |
| Male | 1.00 | 1.42 (1.17-1.72) | 2.11 (1.01-4.40) | 1.90 (1.48-2.43) | 2.29 (1.45-3.62) |
| Female | 1.00 | 1.18 (0.96-1.45) | 2.01 (1.10-3.66) | 1.48 (1.17-1.88) | 1.47 (1.01-2.16) |
| The *P* value of interaction between gender group and Weight change patterns | - | 0.2047 | 0.9207 | 0.3607 | 0.2595 |
| **Prevalence of congestive heart failure** |  |  |  |  |  |
| Stratified by age , years |  |  |  |  |  |
| ≤65 | 1.00 | 1.36 (0.85-2.18) | 2.52 (1.16-5.50) | 2.39 (1.60-3.58) | 5.10 (2.89-8.98) |
| ＞65 | 1.00 | 1.18 (0.92-1.51) | 1.89 (0.91-3.93) | 1.99 (1.48-2.69) | 2.41 (1.39-4.18) |
| The *P* value of interaction between age group and Weight change patterns | - | 0.6676 | 0.4689 | 0.4531 | 0.0495 |
| Stratified by gender |  |  |  |  |  |
| Male | 1.00 | 1.52 (1.16-2.00) | 1.95 (1.03-3.67) | 2.72 (1.91-3.86) | 4.40 (2.40-8.08) |
| Female | 1.00 | 0.98 (0.68-1.41) | 2.81 (1.24-6.34) | 1.64 (1.19-2.26) | 3.13 (2.00-4.92) |
| The *P* value of interaction between gender group and Weight change patterns | - | 0.0443 | 0.4552 | 0.0250 | 0.4407 |

**Continuous Table S3 | Odds ratio (95% CIs) of CVD with weight change patterns stratified by age and gender*.**

|  | **Weight change patterns** | | | | |
| --- | --- | --- | --- | --- | --- |
|  | **Stable normal** | **Maximum overweight** | **Obese to non-obese** | **Non-obese to obese** | **Stable obese** |
| **Prevalence of coronary heart disease** |  |  |  |  |  |
| Stratified by age , years |  |  |  |  |  |
| ≤65 | 1.00 | 1.13 (0.73-1.76) | 2.89 (1.36-6.16) | 1.52 (0.92-2.50) | 2.25 (1.16-4.38) |
| ＞65 | 1.00 | 1.14 (0.91-1.43) | 1.25 (0.61-2.55) | 1.32 (0.97-1.79) | 1.71 (0.91-3.23) |
| The *P* value of interaction between age group and Weight change patterns | - | 0.7859 | 0.1125 | 0.9195 | 0.6799 |
| Stratified by gender |  |  |  |  |  |
| Male | 1.00 | 1.23 (0.97-1.58) | 1.65 (0.86-3.14) | 1.48 (1.03-2.12) | 2.68 (1.55-4.64) |
| Female | 1.00 | 0.97 (0.68-1.37) | 3.27 (1.44-7.43) | 1.36 (0.93-1.98) | 1.08 (0.36-3.24) |
| The *P* value of interaction between gender group and Weight change patterns | - | 0.2170 | 0.1701 | 0.7409 | 0.1553 |
| **Prevalence of angina pectoris** |  |  |  |  |  |
| Stratified by age , years |  |  |  |  |  |
| ≤65 | 1.00 | 0.96 (0.62-1.49) | 1.91 (0.74-4.91) | 1.37 (0.93-2.03) | 1.85 (0.87-3.94) |
| ＞65 | 1.00 | 1.31 (0.98-1.74) | 1.03 (0.40-2.70) | 1.69 (1.24-2.30) | 1.57 (0.74-3.36) |
| The *P* value of interaction between age group and Weight change patterns | - | 0.1897 | 0.3274 | 0.4729 | 0.6846 |
| Stratified by gender |  |  |  |  |  |
| Male | 1.00 | 1.12 (0.73-1.74) | 1.90 (0.83-4.34) | 1.72 (1.13-2.61) | 2.64 (1.24-5.61) |
| Female | 1.00 | 1.20 (0.79-1.81) | 1.06 (0.20-5.57) | 1.31 (0.90-1.91) | 1.24 (0.48-3.17) |
| The *P* value of interaction between gender group and Weight change patterns | - | 0.8299 | 0.6568 | 0.5685 | 0.3006 |
| **Prevalence of heart attack** |  |  |  |  |  |
| Stratified by age , years |  |  |  |  |  |
| ≤65 | 1.00 | 1.35 (0.98-1.88) | 5.09 (2.01-12.90) | 1.84 (1.27-2.69) | 2.96 (1.67-5.22) |
| ＞65 | 1.00 | 1.28 (1.02-1.62) | 1.60 (0.82-3.11) | 1.36 (0.99-1.87) | 1.18 (0.65-2.14) |
| The *P* value of interaction between age group and Weight change patterns | - | 0.9712 | 0.0144 | 0.1407 | 0.0022 |
| Stratified by gender |  |  |  |  |  |

**Continuous Table S3 | Odds ratio (95% CIs) of CVD with weight change patterns stratified by age and gender*.**

|  | **Weight change patterns** | | | | |
| --- | --- | --- | --- | --- | --- |
|  | **Stable normal** | **Maximum overweight** | **Obese to non-obese** | **Non-obese to obese** | **Stable obese** |
| Male | 1.00 | 1.46 (1.12-1.89) | 3.74 (1.49-9.39) | 1.87 (1.39-2.51) | 2.92 (1.70-5.01) |
| Female | 1.00 | 1.17 (0.83-1.65) | 2.42 (0.97-6.08) | 1.25 (0.85-1.84) | 1.42 (0.71-2.87) |
| The *P* value of interaction between gender group and Weight change patterns | - | 0.3066 | 0.6134 | 0.1651 | 0.1639 |
| **Prevalence of stroke** |  |  |  |  |  |
| Stratified by age , years |  |  |  |  |  |
| ≤65 | 1.00 | 1.05 (0.74-1.51) | 1.18 (0.54-2.57) | 1.47 (1.04-2.08) | 1.58 (0.87-2.84) |
| ＞65 | 1.00 | 1.01 (0.80-1.28) | 2.23 (1.04-4.80) | 1.07 (0.83-1.39) | 0.86 (0.48-1.51) |
| The *P* value of interaction between age group and Weight change patterns | - | 0.9690 | 0.3140 | 0.1225 | 0.1288 |
| Stratified by gender |  |  |  |  |  |
| Male | 1.00 | 1.08 (0.79-1.47) | 1.54 (0.74-3.21) | 1.42 (1.06-1.90) | 1.62 (0.76-3.46) |
| Female | 1.00 | 1.01 (0.78-1.31) | 1.89 (0.81-4.41) | 1.13 (0.86-1.48) | 1.07 (0.67-1.72) |
| The *P* value of interaction between gender group and Weight change patterns | - | 0.8399 | 0.7646 | 0.4707 | 0.4873 |
| **From 10 years before baseline to baseline** |  |  |  |  |  |
| **Prevalence of cardiovascular diseases** |  |  |  |  |  |
| Stratified by age , years |  |  |  |  |  |
| ≤65 | 1.00 | 1.31 (0.99-1.73) | 2.14 (1.47-3.12) | 1.84 (1.35-2.50) | 2.24 (1.69-2.97) |
| ＞65 | 1.00 | 1.22 (1.04-1.43) | 2.00 (1.53-2.63) | 1.69 (1.26-2.27) | 1.51 (1.23-1.85) |
| The *P* value of interaction between age group and Weight change patterns | - | 0.9289 | 0.9621 | 0.7514 | 0.0413 |
| Stratified by gender |  |  |  |  |  |
| Male | 1.00 | 1.35 (1.07-1.72) | 2.11 (1.57-2.86) | 1.83 (1.30-2.57) | 2.11 (1.60-2.78) |
| Female | 1.00 | 1.15 (0.90-1.47) | 2.11 (1.51-2.94) | 1.59 (1.20-2.11) | 1.58 (1.21-2.07) |
| The *P* value of interaction between gender group and Weight change patterns | - | 0.3272 | 0.9432 | 0.9596 | 0.2660 |
| **Prevalence of congestive heart failure** |  |  |  |  |  |
| Stratified by age , years |  |  |  |  |  |
| ≤65 | 1.00 | 1.06 (0.64-1.75) | 2.35 (1.28-4.29) | 1.93 (1.17-3.18) | 3.10 (1.98-4.86) |

**Continuous Table S3 | Odds ratio (95% CIs) of CVD with weight change patterns stratified by age and gender*.**

|  | **Weight change patterns** | | | | |
| --- | --- | --- | --- | --- | --- |
|  | **Stable normal** | **Maximum overweight** | **Obese to non-obese** | **Non-obese to obese** | **Stable obese** |
| ＞65 | 1.00 | 1.24 (0.92-1.67) | 2.52 (1.65-3.86) | 2.44 (1.62-3.67) | 2.34 (1.72-3.18) |
| The *P* value of interaction between age group and Weight change patterns | - | 0.5385 | 0.9242 | 0.4726 | 0.2568 |
| Stratified by gender |  |  |  |  |  |
| Male | 1.00 | 1.47 (1.05-2.07) | 2.51 (1.57-4.03) | 2.86 (1.78-4.61) | 3.26 (2.27-4.68) |
| Female | 1.00 | 0.90 (0.59-1.37) | 2.47 (1.37-4.42) | 1.61 (1.04-2.50) | 2.20 (1.47-3.29) |
| The *P* value of interaction between gender group and Weight change patterns | - | 0.0504 | 0.9696 | 0.0806 | 0.1738 |
| **Prevalence of coronary heart disease** |  |  |  |  |  |
| Stratified by age , years |  |  |  |  |  |
| ≤65 | 1.00 | 1.29 (0.80-2.05) | 1.54 (0.78-3.04) | 1.43 (0.76-2.71) | 2.00 (1.18-3.32) |
| ＞65 | 1.00 | 0.97 (0.78-1.22) | 1.49 (1.01-2.20) | 1.72 (1.13-2.63) | 1.10 (0.79-1.54) |
| The *P* value of interaction between age group and Weight change patterns | - | 0.4293 | 0.9477 | 0.4839 | 0.1273 |
| Stratified by gender |  |  |  |  |  |
| Male | 1.00 | 1.12 (0.84-1.50) | 1.56 (1.07-2.28) | 1.35 (0.85-2.14) | 1.68 (1.14-2.47) |
| Female | 1.00 | 0.98 (0.64-1.52) | 1.46 (0.77-2.75) | 1.80 (1.03-3.15) | 1.13 (0.73-1.74) |
| The *P* value of interaction between gender group and Weight change patterns | - | 0.5873 | 0.8296 | 0.3829 | 0.1754 |
| **Prevalence of angina pectoris** |  |  |  |  |  |
| Stratified by age , years |  |  |  |  |  |
| ≤65 | 1.00 | 1.10 (0.70-1.72) | 0.76 (0.41-1.42) | 1.17 (0.70-1.94) | 1.72 (1.10-2.68) |
| ＞65 | 1.00 | 1.21 (0.90-1.62) | 1.63 (1.04-2.55) | 1.97 (1.21-3.20) | 1.56 (1.10-2.21) |
| The *P* value of interaction between age group and Weight change patterns | - | 0.7005 | 0.0532 | 0.1530 | 0.5530 |
| Stratified by gender |  |  |  |  |  |
| Male | 1.00 | 1.07 (0.67-1.74) | 0.90 (0.52-1.56) | 1.23 (0.70-2.17) | 2.00 (1.21-3.30) |
| Female | 1.00 | 1.21 (0.76-1.94) | 1.71 (0.84-3.48) | 1.65 (1.01-2.69) | 1.23 (0.75-2.01) |
| The *P* value of interaction between gender group and Weight change patterns | - | 0.7458 | 0.1706 | 0.2364 | 0.2968 |

**Continuous Table S3 | Odds ratio (95% CIs) of CVD with weight change patterns stratified by age and gender*.**

|  | **Weight change patterns** | | | | |
| --- | --- | --- | --- | --- | --- |
|  | **Stable normal** | **Maximum overweight** | **Obese to non-obese** | **Non-obese to obese** | **Stable obese** |
| **Prevalence of heart attack** |  |  |  |  |  |
| Stratified by age , years |  |  |  |  |  |
| ≤65 | 1.00 | 1.24 (0.84-1.83) | 2.27 (1.41-3.65) | 1.60 (0.99-2.57) | 2.23 (1.47-3.40) |
| ＞65 | 1.00 | 1.07 (0.82-1.39) | 2.01 (1.41-2.88) | 1.70 (1.11-2.61) | 1.14 (0.81-1.61) |
| The *P* value of interaction between age group and Weight change patterns | - | 0.7083 | 0.7722 | 0.8035 | 0.0237 |
| Stratified by gender |  |  |  |  |  |
| Male | 1.00 | 1.25 (0.93-1.68) | 2.56 (1.64-3.97) | 1.68 (1.13-2.50) | 2.09 (1.50-2.92) |
| Female | 1.00 | 1.00 (0.65-1.53) | 1.75 (1.20-2.54) | 1.47 (0.91-2.37) | 1.09 (0.71-1.70) |
| The *P* value of interaction between gender group and Weight change patterns | - | 0.3956 | 0.1517 | 0.9441 | 0.0322 |
| **Prevalence of stroke** |  |  |  |  |  |
| Stratified by age , years |  |  |  |  |  |
| ≤65 | 1.00 | 0.95 (0.64-1.42) | 2.14 (1.27-3.59) | 1.41 (0.93-2.15) | 1.68 (1.09-2.60) |
| ＞65 | 1.00 | 1.16 (0.90-1.50) | 1.67 (1.11-2.51) | 1.15 (0.76-1.76) | 1.23 (0.93-1.64) |
| The *P* value of interaction between age group and Weight change patterns | - | 0.3350 | 0.4896 | 0.4209 | 0.20545 |
| Stratified by gender |  |  |  |  |  |
| Male | 1.00 | 1.21 (0.82-1.77) | 2.05 (1.28-3.29) | 1.58 (0.96-2.60) | 1.74 (1.19-2.57) |
| Female | 1.00 | 1.03 (0.77-1.37) | 1.80 (1.15-2.83) | 1.15 (0.81-1.65) | 1.26 (0.92-1.73) |
| The *P* value of interaction between gender group and Weight change patterns | - | 0.5711 | 0.6853 | 0.5575 | 0.2699 |

Abbreviations: 95% CI, 95% Confidence Interval; CVD, Cardiovascular diseases.

*All the estimated values have taken into account the complex sampling designs. Among 35998 participants, a total of 1512, 964, and 1001 participants were excluded in analyses from age 25 years to 10 years before baseline, age 25 years to baseline, and 10 years before baseline to baseline, respectively, owing to missing values of body mass index at both times. Model adjusted for age, gender, race/ethnicity, educational level, income level, marital status, alcohol consumption, smoking status, family history of diabetes, family history of heart disease, moderate exercise status and general health condition.

**Table S4 | Odds ratio (95% CIs) of CVD with absolute weight change groups across adulthood***

|  | **Absolute weight change groups** | | | | |
| --- | --- | --- | --- | --- | --- |
|  | **Weight loss ≥2.5 kg** | **Weight change within 2.5 kg** | **Weight gain ≥2 .5 kg and <10.0 kg** | **Weight gain**  **≥10 kg and <20 kg** | **Weight gain ≥20 kg** |
| **From age 25 years to 10 years before baseline** |  |  |  |  |  |
| Prevalence of cardiovascular diseases |  |  |  |  |  |
| Model 1 | 1.08 (0.89-1.31) | 1.00 | 0.89 (0.80-0.99) | 1.18 (1.06-1.32) | 1.65 (1.49-1.84) |
| Model 2 | 1.01 (0.76-1.33) | 1.00 | 0.88 (0.75-1.03) | 1.13 (0.97-1.32) | 1.64 (1.41-1.90) |
| Model 3 | 1.00 (0.76-1.31) | 1.00 | 0.91 (0.78-1.07) | 1.10 (0.93-1.29) | 1.51 (1.29-1.76) |
| Prevalence of congestive heart failure |  |  |  |  |  |
| Model 1 | 1.41 (1.04-1.91) | 1.00 | 0.79 (0.66-0.95) | 1.02 (0.84-1.24) | 1.96 (1.60-2.39) |
| Model 2 | 1.57 (1.01-2.45) | 1.00 | 0.88 (0.66-1.18) | 1.04 (0.79-1.37) | 2.13 (1.63-2.79) |
| Model 3 | 1.60 (1.04-2.47) | 1.00 | 0.94 (0.70-1.27) | 1.02 (0.77-1.34) | 1.97 (1.51-2.57) |
| Prevalence of coronary heart disease |  |  |  |  |  |
| Model 1 | 1.22 (0.92-1.61) | 1.00 | 0.99 (0.84-1.17) | 1.32 (1.10-1.58) | 1.52 (1.28-1.81) |
| Model 2 | 1.02 (0.67-1.55) | 1.00 | 0.89 (0.72-1.10) | 1.05 (0.84-1.33) | 1.21 (0.98-1.49) |
| Model 3 | 1.02 (0.67-1.55) | 1.00 | 0.90 (0.73-1.12) | 1.00 (0.79-1.26) | 1.10 (0.89-1.35) |
| Prevalence of angina pectoris |  |  |  |  |  |
| Model 1 | 1.37 (0.98-1.90) | 1.00 | 0.96 (0.77-1.20) | 1.51 (1.23-1.85) | 1.80 (1.45-2.23) |
| Model 2 | 1.03 (0.61-1.74) | 1.00 | 0.80 (0.59-1.08) | 1.25 (0.93-1.69) | 1.64 (1.23-2.17) |
| Model 3 | 1.06 (0.63-1.79) | 1.00 | 0.83 (0.61-1.12) | 1.22 (0.90-1.66) | 1.50 (1.13-2.00) |
| Prevalence of heart attack |  |  |  |  |  |
| Model 1 | 1.30 (0.99-1.71) | 1.00 | 0.91 (0.78-1.06) | 1.23 (1.03-1.46) | 1.63 (1.38-1.91) |
| Model 2 | 1.44 (1.01-2.07) | 1.00 | 0.89 (0.72-1.10) | 1.10 (0.86-1.40) | 1.51 (1.23-1.86) |
| Model 3 | 1.46 (1.02-2.09) | 1.00 | 0.93 (0.76-1.13) | 1.09 (0.84-1.41) | 1.42 (1.14-1.77) |
| Prevalence of stroke |  |  |  |  |  |
| Model 1 | 0.87 (0.67-1.13) | 1.00 | 0.79 (0.68-0.93) | 0.92 (0.77-1.11) | 1.37 (1.18-1.59) |
| Model 2 | 0.76 (0.52-1.10) | 1.00 | 0.73 (0.58-0.92) | 0.88 (0.68-1.15) | 1.30 (1.03-1.64) |
| Model 3 | 0.77 (0.53-1.11) | 1.00 | 0.75 (0.60-0.95) | 0.86 (0.66-1.13) | 1.21 (0.95-1.53) |
| **From age 25 years to baseline** |  |  |  |  |  |
| Prevalence of cardiovascular diseases |  |  |  |  |  |
| Model 1 | 1.36 (1.17-1.52) | 1.00 | 0.92 (0.80-1.05) | 1.11 (0.99-1.24) | 1.53 (1.36-1.73) |
| Model 2 | 1.37 (1.12-1.68) | 1.00 | 1.03 (0.86-1.23) | 1.11 (0.95-1.30) | 1.56 (1.32-1.85) |

**Continuous Table S4 | Odds ratio (95% CIs) of CVD with absolute weight change groups across adulthood***

|  | **Absolute weight change groups** | | | | |
| --- | --- | --- | --- | --- | --- |
|  | **Weight loss ≥2.5 kg** | **Weight change within 2.5 kg** | **Weight gain ≥2 .5 kg and <10.0 kg** | **Weight gain**  **≥10 kg and <20 kg** | **Weight gain ≥20 kg** |
| Model 3 | 1.28 (1.02-1.59) | 1.00 | 1.07 (0.89-1.28) | 1.13 (0.96-1.33) | 1.35 (1.14-1.61) |
| Prevalence of congestive heart failure |  |  |  |  |  |
| Model 1 | 1.55 (1.23-1.96) | 1.00 | 0.80 (0.65-0.99) | 0.95 (0.77-1.18) | 1.71 (1.41-2.08) |
| Model 2 | 1.54 (1.11-2.13) | 1.00 | 0.95 (0.72-1.25) | 1.15 (0.88-1.50) | 1.86 (1.47-2.35) |
| Model 3 | 1.43 (1.02-2.00) | 1.00 | 0.98 (0.74-1.30) | 1.20 (0.92-1.57) | 1.64 (1.29-2.09) |
| Prevalence of coronary heart disease |  |  |  |  |  |
| Model 1 | 1.34 (1.09-1.64) | 1.00 | 1.00 (0.82-1.21) | 1.23 (1.02-1.48) | 1.38 (1.14-1.66) |
| Model 2 | 1.32 (0.97-1.78) | 1.00 | 0.95 (0.75-1.20) | 1.05 (0.81-1.37) | 1.22 (0.95-1.57) |
| Model 3 | 1.26 (0.92-1.73) | 1.00 | 0.98 (0.77-1.25) | 1.06 (0.81-1.38) | 1.05 (0.82-1.35) |
| Prevalence of angina pectoris |  |  |  |  |  |
| Model 1 | 1.37 (1.03-1.82) | 1.00 | 1.01 (0.79-1.30) | 1.33 (1.06-1.66) | 1.92 (1.55-2.39) |
| Model 2 | 1.27 (0.85-1.91) | 1.00 | 0.87 (0.61-1.25) | 1.13 (0.82-1.56) | 1.61 (1.19-2.17) |
| Model 3 | 1.15 (0.75-1.76) | 1.00 | 0.92 (0.64-1.31) | 1.13 (0.82-1.57) | 1.36 (1.01-1.84) |
| Prevalence of heart attack |  |  |  |  |  |
| Model 1 | 1.59 (1.29-1.97) | 1.00 | 1.10 (0.90-1.36) | 1.29 (1.07-1.55) | 1.50 (1.23-1.83) |
| Model 2 | 1.77 (1.30-2.41) | 1.00 | 1.26 (0.97-1.63) | 1.30 (0.99-1.71) | 1.55 (1.15-2.09) |
| Model 3 | 1.68 (1.21-2.34) | 1.00 | 1.35 (1.04-1.75) | 1.32 (0.98-1.76) | 1.39 (1.03-1.87) |
| Prevalence of stroke |  |  |  |  |  |
| Model 1 | 1.27 (1.02-1.57) | 1.00 | 0.88 (0.72-1.07) | 0.98 (0.81-1.19) | 1.28 (1.06-1.54) |
| Model 2 | 1.26 (0.94-1.68) | 1.00 | 0.99 (0.78-1.26) | 0.96 (0.74-1.24) | 1.30 (1.01-1.69) |
| Model 3 | 1.21 (0.88-1.65) | 1.00 | 1.04 (0.81-1.34) | 1.00 (0.76-1.31) | 1.17 (0.89-1.53) |
| **From 10 years before baseline to baseline** |  |  |  |  |  |
| Prevalence of cardiovascular diseases |  |  |  |  |  |
| Model 1 | 1.81 (1.61-2.02) | 1.00 | 1.15 (1.01-1.31) | 1.83 (1.58-2.11) | 2.66 (2.22-3.18) |
| Model 2 | 1.58 (1.36-1.84) | 1.00 | 1.04 (0.87-1.23) | 1.62 (1.33-1.97) | 2.06 (1.59-2.67) |
| Model 3 | 1.39 (1.19-1.64) | 1.00 | 0.98 (0.82-1.16) | 1.33 (1.08-1.64) | 1.50 (1.16-1.94) |
| Prevalence of congestive heart failure |  |  |  |  |  |
| Model 1 | 2.09 (1.76-2.48) | 1.00 | 1.20 (0.99-1.46) | 1.83 (1.38-2.42) | 3.89 (3.00-5.04) |
| Model 2 | 2.00 (1.61-2.50) | 1.00 | 1.22 (0.94-1.58) | 1.88 (1.31-2.70) | 3.19 (2.24-4.53) |

**Continuous Table S4 | Odds ratio (95% CIs) of CVD with absolute weight change groups across adulthood***

|  | **Absolute weight change groups** | | | | |
| --- | --- | --- | --- | --- | --- |
|  | **Weight loss ≥2.5 kg** | **Weight change within 2.5 kg** | **Weight gain ≥2 .5 kg and <10.0 kg** | **Weight gain**  **≥10 kg and <20 kg** | **Weight gain ≥20 kg** |
| Model 3 | 1.73 (1.36-2.20) | 1.00 | 1.17 (0.90-1.52) | 1.52 (1.05-2.22) | 2.31 (1.62-3.32) |
| Prevalence of coronary heart disease |  |  |  |  |  |
| Model 1 | 1.56 (1.33-1.83) | 1.00 | 1.27 (1.06-1.53) | 1.60 (1.29-2.00) | 2.07 (1.55-2.77) |
| Model 2 | 1.28 (1.03-1.59) | 1.00 | 1.07 (0.82-1.38) | 1.33 (0.97-1.81) | 1.78 (1.16-2.72) |
| Model 3 | 1.17 (0.93-1.46) | 1.00 | 1.01 (0.77-1.31) | 1.09 (0.78-1.53) | 1.30 (0.85-1.99) |
| Prevalence of angina pectoris |  |  |  |  |  |
| Model 1 | 1.51 (1.22-1.88) | 1.00 | 1.23 (1.01-1.49) | 1.87 (1.44-2.43) | 2.44 (1.75-3.40) |
| Model 2 | 1.29 (0.96-1.74) | 1.00 | 1.00 (0.75-1.32) | 1.31 (0.90-1.89) | 1.82 (1.16-2.88) |
| Model 3 | 1.14 (0.84-1.53) | 1.00 | 0.95 (0.72-1.25) | 1.04 (0.70-1.53) | 1.28 (0.81-2.02) |
| Prevalence of heart attack |  |  |  |  |  |
| Model 1 | 1.69 (1.45-1.98) | 1.00 | 1.16 (0.95-1.40) | 1.63 (1.32-2.01) | 1.97 (1.51-2.59) |
| Model 2 | 1.35 (1.10-1.65) | 1.00 | 1.03 (0.80-1.34) | 1.34 (1.04-1.74) | 1.59 (1.07-2.38) |
| Model 3 | 1.21 (0.99-1.49) | 1.00 | 0.99 (0.76-1.28) | 1.09 (0.82-1.46) | 1.21 (0.83-1.76) |
| Prevalence of stroke |  |  |  |  |  |
| Model 1 | 1.94 (1.65-2.28) | 1.00 | 1.03 (0.85-1.25) | 1.85 (1.51-2.28) | 2.20 (1.73-2.80) |
| Model 2 | 1.83 (1.47-2.27) | 1.00 | 1.03 (0.79-1.34) | 1.84 (1.38-2.44) | 1.66 (1.16-2.38) |
| Model 3 | 1.65 (1.32-2.07) | 1.00 | 0.99 (0.76-1.28) | 1.58 (1.17-2.12) | 1.29 (0.91-1.82) |

Abbreviations: 95% CI, 95% Confidence Interval; CVD, Cardiovascular diseases.

*All the estimated values have taken into account the complex sampling designs. Among 35998 participants, a total of 1512, 964, and 1001 participants were excluded in analyses from age 25 years to 10 years before baseline, age 25 years to baseline, and 10 years before baseline to baseline, respectively, owing to missing values of body mass index at both times. Model 1 adjusted for baseline age, gender and race/ethnicity. Model 2 further adjusted for educational level, income level, marital status, alcohol consumption, smoking status, family history of diabetes, and family history of heart disease. Model 3 further adjusted for baseline moderate exercise status and general health condition.

**Table S5 | Odds ratio (95% CIs) of CVD with absolute weight change stratified by age and gender***

|  | **Absolute weight change groups** | | | | |
| --- | --- | --- | --- | --- | --- |
|  | **Weight loss ≥2.5 kg** | **Weight change within 2.5 kg** | **Weight gain**  **≥2 .5 kg and <10.0 kg** | **Weight gain**  **≥10 kg and <20 kg** | **Weight gain ≥20 kg** |
| **From age 25 years to 10 years before baseline** |  |  |  |  |  |
| **Prevalence of cardiovascular diseases** |  |  |  |  |  |
| Stratified by age , years |  |  |  |  |  |
| ≤65 | 1.14 (0.80-1.62) | 1.00 | 0.80 (0.64-1.00) | 1.20 (0.96-1.50) | 1.67 (1.33-2.11) |
| ＞65 | 0.83 (0.59-1.17) | 1.00 | 1.05 (0.85-1.31) | 1.00 (0.81-1.25) | 1.37 (1.12-1.68) |
| The *P* value of interaction between age group and Weight change patterns | 0.2071 | - | 0.0270 | 0.4505 | 0.3420 |
| Stratified by gender |  |  |  |  |  |
| Male | 1.07 (0.75-1.52) | 1.00 | 0.97 (0.76-1.23) | 1.08 (0.84-1.38) | 1.64 (1.32-2.04) |
| Female | 0.91 (0.61-1.35) | 1.00 | 0.84 (0.67-1.06) | 1.09 (0.87-1.37) | 1.39 (1.09-1.78) |
| The *P* value of interaction between gender group and Weight change patterns | 0.4995 | - | 0.2530 | 0.6421 | 0.1608 |
| **Prevalence of congestive heart failure** |  |  |  |  |  |
| Stratified by age , years |  |  |  |  |  |
| ≤65 | 1.47 (0.73-2.95) | 1.00 | 0.82 (0.53-1.26) | 1.13 (0.76-1.67) | 2.29 (1.60-3.27) |
| ＞65 | 1.53 (0.94-2.51) | 1.00 | 0.94 (0.67-1.32) | 0.84 (0.59-1.21) | 1.54 (1.12-2.13) |
| The *P* value of interaction between age group and Weight change patterns | 0.8139 | - | 0.3821 | 0.4094 | 0.1403 |
| Stratified by gender |  |  |  |  |  |
| Male | 1.33 (0.71-2.49) | 1.00 | 0.93 (0.66-1.30) | 0.85 (0.60-1.19) | 1.85 (1.34-2.54) |
| Female | 1.86 (1.01-3.43) | 1.00 | 0.83 (0.52-1.32) | 1.09 (0.69-1.74) | 1.86 (1.23-2.82) |
| The *P* value of interaction between gender group and Weight change patterns | 0.5028 | - | 0.5706 | 0.5329 | 0.8676 |
| **Prevalence of coronary heart disease** |  |  |  |  |  |
| Stratified by age , years |  |  |  |  |  |
| ≤65 | 1.27 (0.65-2.47) | 1.00 | 0.88 (0.62-1.25) | 0.97 (0.63-1.49) | 1.29 (0.91-1.84) |
| ＞65 | 0.85 (0.53-1.37) | 1.00 | 0.94 (0.72-1.23) | 1.01 (0.74-1.36) | 0.95 (0.71-1.29) |
| The *P* value of interaction between age group and Weight change patterns | 0.3082 | - | 0.5159 | 0.7359 | 0.3023 |

**Continuous Table S5 | Odds ratio (95% CIs) of CVD with absolute weight change stratified by age and gender***

|  | **Absolute weight change groups** | | | | |
| --- | --- | --- | --- | --- | --- |
|  | **Weight loss ≥2.5 kg** | **Weight change within 2.5 kg** | **Weight gain**  **≥2 .5 kg and <10.0 kg** | **Weight gain**  **≥10 kg and <20 kg** | **Weight gain ≥20 kg** |
| Stratified by gender |  |  |  |  |  |
| Male | 1.13 (0.68-1.89) | 1.00 | 0.96 (0.73-1.27) | 1.00 (0.75-1.33) | 1.25 (0.96-1.62) |
| Female | 0.88 (0.42-1.85) | 1.00 | 0.86 (0.56-1.33) | 1.03 (0.67-1.58) | 1.00 (0.65-1.55) |
| The *P* value of interaction between gender group and Weight change patterns | 0.5816 | - | 0.5263 | 0.9628 | 0.3577 |
| **Prevalence of angina pectoris** |  |  |  |  |  |
| Stratified by age , years |  |  |  |  |  |
| ≤65 | 1.28 (0.63-2.60) | 1.00 | 0.85 (0.52-1.38) | 1.37 (0.83-2.26) | 1.37 (0.86-2.17) |
| ＞65 | 0.81 (0.39-1.67) | 1.00 | 0.81 (0.52-1.26) | 1.04 (0.72-1.49) | 1.55 (1.11-2.16) |
| The *P* value of interaction between age group and Weight change patterns | 0.3901 | - | 0.9216 | 0.4585 | 0.7065 |
| Stratified by gender |  |  |  |  |  |
| Male | 0.98 (0.47-2.03) | 1.00 | 0.89 (0.58-1.35) | 1.21 (0.80-1.83) | 1.61 (1.10-2.35) |
| Female | 1.19 (0.56-2.53) | 1.00 | 0.75 (0.47-1.19) | 1.18 (0.75-1.88) | 1.38 (0.91-2.10) |
| The *P* value of interaction between gender group and Weight change patterns | 0.7337 | - | 0.5410 | 0.8760 | 0.5601 |
| **Prevalence of heart attack** |  |  |  |  |  |
| Stratified by age , years |  |  |  |  |  |
| ≤65 | 1.72 (1.06-2.80) | 1.00 | 0.87 (0.63-1.22) | 1.06 (0.70-1.59) | 1.49 (1.04-2.15) |
| ＞65 | 1.14 (0.72-1.80) | 1.00 | 0.98 (0.74-1.31) | 1.08 (0.81-1.45) | 1.30 (0.97-1.74) |
| The *P* value of interaction between age group and Weight change patterns | 0.2466 | - | 0.5021 | 0.8780 | 0.4790 |
| Stratified by gender |  |  |  |  |  |
| Male | 1.36 (0.84-2.20) | 1.00 | 0.88 (0.66-1.17) | 1.08 (0.81-1.45) | 1.43 (1.05-1.96) |
| Female | 1.64 (0.91-2.95) | 1.00 | 1.04 (0.71-1.51) | 1.09 (0.70-1.68) | 1.45 (1.02-2.07) |
| The *P* value of interaction between gender group and Weight change patterns | 0.6458 | - | 0.6936 | 0.7960 | 0.7972 |
| **Prevalence of stroke** |  |  |  |  |  |
| Stratified by age , years |  |  |  |  |  |

**Continuous Table S5 | Odds ratio (95% CIs) of CVD with absolute weight change stratified by age and gender***

|  | **Absolute weight change groups** | | | | |
| --- | --- | --- | --- | --- | --- |
|  | **Weight loss ≥2.5 kg** | **Weight change within 2.5 kg** | **Weight gain**  **≥2 .5 kg and <10.0 kg** | **Weight gain**  **≥10 kg and <20 kg** | **Weight gain ≥20 kg** |
| **Prevalence of stroke** |  |  |  |  |  |
| Stratified by age , years |  |  |  |  |  |
| ≤65 | 0.67 (0.37-1.23) | 1.00 | 0.59 (0.42-0.84) | 0.96 (0.65-1.40) | 1.39 (0.98-1.97) |
| ＞65 | 0.86 (0.53-1.40) | 1.00 | 0.97 (0.70-1.36) | 0.83 (0.58-1.19) | 1.18 (0.85-1.65) |
| The *P* value of interaction between age group and Weight change patterns | 0.5327 | - | 0.0268 | 0.7712 | 0.6156 |
| Stratified by gender |  |  |  |  |  |
| Male | 0.76 (0.45-1.29) | 1.00 | 0.87 (0.59-1.28) | 0.93 (0.62-1.39) | 1.47 (1.09-1.99) |
| Female | 0.78 (0.47-1.27) | 1.00 | 0.69 (0.51-0.92) | 0.84 (0.60-1.16) | 1.09 (0.77-1.52) |
| The *P* value of interaction between gender group and Weight change patterns | 0.9398 | - | 0.3636 | 0.6584 | 0.1729 |
| **From age 25 years to baseline** |  |  |  |  |  |
| **Prevalence of cardiovascular diseases** |  |  |  |  |  |
| Stratified by age , years |  |  |  |  |  |
| ≤65 | 1.83 (1.31-2.56) | 1.00 | 1.17 (0.86-1.59) | 1.13 (0.85-1.49) | 1.66 (1.25-2.22) |
| ＞65 | 0.91 (0.70-1.17) | 1.00 | 1.02 (0.80-1.29) | 1.15 (0.92-1.43) | 1.09 (0.86-1.37) |
| The *P* value of interaction between age group and Weight change patterns | 0.0003 | - | 0.7259 | 0.7876 | 0.0259 |
| Stratified by gender |  |  |  |  |  |
| Male | 1.27 (0.92-1.73) | 1.00 | 0.99 (0.78-1.26) | 1.07 (0.85-1.34) | 1.23 (0.97-1.56) |
| Female | 1.27 (0.91-1.77) | 1.00 | 1.16 (0.88-1.54) | 1.19 (0.91-1.56) | 1.46 (1.13-1.88) |
| The *P* value of interaction between gender group and Weight change patterns | 0.8630 | - | 0.4999 | 0.6138 | 0.2146 |
| **Prevalence of congestive heart failure** |  |  |  |  |  |
| Stratified by age , years |  |  |  |  |  |
| ≤65 | 3.05 (1.69-5.53) | 1.00 | 1.71 (0.85-3.41) | 2.14 (1.19-3.83) | 2.78 (1.64-4.70) |
| ＞65 | 1.01 (0.68-1.50) | 1.00 | 0.78 (0.56-1.10) | 0.93 (0.66-1.30) | 1.27 (0.93-1.73) |
| The *P* value of interaction between age group and Weight change patterns | 0.0014 | - | 0.1235 | 0.0451 | 0.0233 |

**Continuous Table S5 | Odds ratio (95% CIs) of CVD with absolute weight change stratified by age and gender***

|  | **Absolute weight change groups** | | | | |
| --- | --- | --- | --- | --- | --- |
|  | **Weight loss ≥2.5 kg** | **Weight change within 2.5 kg** | **Weight gain**  **≥2 .5 kg and <10.0 kg** | **Weight gain**  **≥10 kg and <20 kg** | **Weight gain ≥20 kg** |
| Stratified by gender |  |  |  |  |  |
| Male | 1.81 (1.09-3.00) | 1.00 | 1.13 (0.73-1.76) | 1.53 (0.99-2.36) | 1.96 (1.28-3.00) |
| Female | 1.06 (0.71-1.59) | 1.00 | 0.82 (0.55-1.21) | 0.88 (0.61-1.27) | 1.29 (0.97-1.72) |
| The *P* value of interaction between gender group and Weight change patterns | 0.0986 | - | 0.2059 | 0.0495 | 0.1073 |
| **Prevalence of coronary heart disease** |  |  |  |  |  |
| Stratified by age , years |  |  |  |  |  |
| ≤65 | 1.91 (1.10-3.33) | 1.00 | 1.10 (0.73-1.65) | 1.11 (0.68-1.83) | 1.23 (0.77-1.97) |
| ＞65 | 0.95 (0.64-1.39) | 1.00 | 0.94 (0.68-1.29) | 1.03 (0.76-1.39) | 0.88 (0.61-1.26) |
| The *P* value of interaction between age group and Weight change patterns | 0.0231 | - | 0.7915 | 0.9831 | 0.4346 |
| Stratified by gender |  |  |  |  |  |
| Male | 1.33 (0.86-2.05) | 1.00 | 1.00 (0.74-1.37) | 1.11 (0.81-1.53) | 1.00 (0.74-1.37) |
| Female | 1.09 (0.67-1.76) | 1.00 | 0.91 (0.53-1.55) | 0.93 (0.60-1.45) | 1.11 (0.72-1.71) |
| The *P* value of interaction between gender group and Weight change patterns | 0.7078 | - | 0.6934 | 0.4359 | 0.6949 |
| **Prevalence of angina pectoris** |  |  |  |  |  |
| Stratified by age , years |  |  |  |  |  |
| ≤65 | 1.73 (0.86-3.46) | 1.00 | 0.90 (0.48-1.68) | 1.12 (0.63-1.98) | 1.46 (0.79-2.68) |
| ＞65 | 0.73 (0.44-1.22) | 1.00 | 0.95 (0.60-1.51) | 1.15 (0.75-1.76) | 1.23 (0.81-1.85) |
| The *P* value of interaction between age group and Weight change patterns | 0.0318 | - | 0.7620 | 0.8924 | 0.6022 |
| Stratified by gender |  |  |  |  |  |
| Male | 1.08 (0.63-1.86) | 1.00 | 0.77 (0.48-1.22) | 1.09 (0.66-1.81) | 1.30 (0.85-1.98) |
| Female | 1.24 (0.63-2.42) | 1.00 | 1.22 (0.67-2.20) | 1.20 (0.72-1.99) | 1.43 (0.88-2.31) |
| The *P* value of interaction between gender group and Weight change patterns | 0.6443 | - | 0.2340 | 0.7352 | 0.5900 |
| **Prevalence of heart attack** |  |  |  |  |  |
| Stratified by age , years |  |  |  |  |  |

**Continuous Table S5 | Odds ratio (95% CIs) of CVD with absolute weight change stratified by age and gender***

|  | **Absolute weight change groups** | | | | |
| --- | --- | --- | --- | --- | --- |
|  | **Weight loss ≥2.5 kg** | **Weight change within 2.5 kg** | **Weight gain**  **≥2 .5 kg and <10.0 kg** | **Weight gain**  **≥10 kg and <20 kg** | **Weight gain ≥20 kg** |
| ≤65 | 2.32 (1.31-4.13) | 1.00 | 1.50 (0.93-2.43) | 1.31 (0.76-2.27) | 1.48 (0.89-2.46) |
| ＞65 | 1.27 (0.91-1.79) | 1.00 | 1.25 (0.94-1.67) | 1.32 (0.95-1.84) | 1.25 (0.88-1.79) |
| The *P* value of interaction between age group and Weight change patterns | 0.0529 | - | 0.6895 | 0.9366 | 0.4975 |
| Stratified by gender |  |  |  |  |  |
| Male | 1.74 (1.10-2.77) | 1.00 | 1.29 (0.93-1.78) | 1.49 (1.04-2.12) | 1.26 (0.86-1.85) |
| Female | 1.54 (0.91-2.62) | 1.00 | 1.43 (0.89-2.30) | 1.01 (0.61-1.67) | 1.52 (1.01-2.30) |
| The *P* value of interaction between gender group and Weight change patterns | 0.8303 | - | 0.7351 | 0.2081 | 0.3470 |
| **Prevalence of stroke** |  |  |  |  |  |
| Stratified by age , years |  |  |  |  |  |
| ≤65 | 1.62 (0.96-2.73) | 1.00 | 0.97 (0.58-1.62) | 0.98 (0.62-1.56) | 1.45 (0.93-2.24) |
| ＞65 | 0.96 (0.68-1.35) | 1.00 | 1.12 (0.80-1.56) | 1.04 (0.71-1.51) | 0.99 (0.69-1.40) |
| The *P* value of interaction between age group and Weight change patterns | 0.0596 | - | 0.5353 | 0.8415 | 0.1292 |
| Stratified by gender |  |  |  |  |  |
| Male | 1.00 (0.65-1.52) | 1.00 | 0.98 (0.68-1.40) | 0.79 (0.54-1.16) | 1.16 (0.82-1.63) |
| Female | 1.43 (0.93-2.19) | 1.00 | 1.10 (0.77-1.58) | 1.18 (0.81-1.74) | 1.20 (0.83-1.74) |
| The *P* value of interaction between gender group and Weight change patterns | 0.2025 | - | 0.5293 | 0.0992 | 0.6118 |
| **From 10 years before baseline to baseline** |  |  |  |  |  |
| **Prevalence of cardiovascular diseases** |  |  |  |  |  |
| Stratified by age , years |  |  |  |  |  |
| ≤65 | 1.61 (1.24-2.10) | 1.00 | 0.95 (0.73-1.24) | 1.39 (1.06-1.83) | 1.44 (1.06-1.95) |
| ＞65 | 1.21 (0.98-1.49) | 1.00 | 1.01 (0.81-1.25) | 1.27 (0.95-1.70) | 1.68 (0.99-2.87) |
| The *P* value of interaction between age group and Weight change patterns | 0.0489 | - | 0.8612 | 0.3856 | 0.8680 |
| Stratified by gender |  |  |  |  |  |
| Male | 1.39 (1.12-1.72) | 1.00 | 1.00 (0.78-1.27) | 1.19 (0.88-1.60) | 1.39 (0.98-1.97) |

**Continuous Table S5 | Odds ratio (95% CIs) of CVD with absolute weight change stratified by age and gender***

|  | **Absolute weight change groups** | | | | |
| --- | --- | --- | --- | --- | --- |
|  | **Weight loss ≥2.5 kg** | **Weight change within 2.5 kg** | **Weight gain**  **≥2 .5 kg and <10.0 kg** | **Weight gain**  **≥10 kg and <20 kg** | **Weight gain ≥20 kg** |
| Female | 1.33 (1.07-1.66) | 1.00 | 0.92 (0.71-1.18) | 1.40 (1.08-1.81) | 1.47 (1.05-2.07) |
| The *P* value of interaction between gender group and Weight change patterns | 0.9970 | - | 0.8720 | 0.0624 | 0.1556 |
| **Prevalence of congestive heart failure** |  |  |  |  |  |
| Stratified by age , years |  |  |  |  |  |
| ≤65 | 1.48 (0.96-2.29) | 1.00 | 0.75 (0.46-1.24) | 1.11 (0.63-1.94) | 1.53 (0.96-2.45) |
| ＞65 | 1.54 (1.16-2.03) | 1.00 | 1.28 (0.93-1.77) | 1.63 (1.14-2.33) | 2.95 (1.54-5.62) |
| The *P* value of interaction between age group and Weight change patterns | 0.7648 | - | 0.1009 | 0.2147 | 0.1161 |
| Stratified by gender |  |  |  |  |  |
| Male | 1.57 (1.18-2.08) | 1.00 | 1.12 (0.81-1.56) | 1.38 (0.81-2.35) | 2.13 (1.33-3.40) |
| Female | 1.45 (1.01-2.08) | 1.00 | 0.99 (0.65-1.51) | 1.37 (0.88-2.12) | 1.99 (1.15-3.44) |
| The *P* value of interaction between gender group and Weight change patterns | 0.8267 | - | 0.5408 | 0.9573 | 0.9524 |
| **Prevalence of coronary heart disease** |  |  |  |  |  |
| Stratified by age , years |  |  |  |  |  |
| ≤65 | 1.40 (0.95-2.07) | 1.00 | 0.92 (0.61-1.40) | 1.01 (0.63-1.64) | 1.18 (0.68-2.04) |
| ＞65 | 1.05 (0.79-1.40) | 1.00 | 1.07 (0.78-1.46) | 1.25 (0.83-1.90) | 1.52 (0.72-3.19) |
| The *P* value of interaction between age group and Weight change patterns | 0.1305 | - | 0.5218 | 0.4138 | 0.5208 |
| Stratified by gender |  |  |  |  |  |
| Male | 1.26 (0.97-1.63) | 1.00 | 1.04 (0.79-1.38) | 0.93 (0.62-1.39) | 1.22 (0.74-2.03) |
| Female | 0.94 (0.64-1.39) | 1.00 | 0.92 (0.58-1.45) | 1.34 (0.80-2.24) | 1.34 (0.65-2.78) |
| The *P* value of interaction between gender group and Weight change patterns | 0.3103 | - | 0.6040 | 0.1934 | 0.6730 |
| **Prevalence of angina pectoris** |  |  |  |  |  |
| Stratified by age , years |  |  |  |  |  |
| ≤65 | 1.44 (0.89-2.33) | 1.00 | 1.09 (0.69-1.73) | 1.21 (0.71-2.04) | 1.26 (0.73-2.19) |
| ＞65 | 0.95 (0.69-1.31) | 1.00 | 0.86 (0.60-1.22) | 0.93 (0.53-1.63) | 1.50 (0.64-3.58) |

**Continuous Table S5 | Odds ratio (95% CIs) of CVD with absolute weight change stratified by age and gender***

|  | **Absolute weight change groups** | | | | |
| --- | --- | --- | --- | --- | --- |
|  | **Weight loss ≥2.5 kg** | **Weight change within 2.5 kg** | **Weight gain**  **≥2 .5 kg and <10.0 kg** | **Weight gain**  **≥10 kg and <20 kg** | **Weight gain ≥20 kg** |
| The *P* value of interaction between age group and Weight change patterns | 0.0387 | - | 0.3859 | 0.3374 | 0.8571 |
| Stratified by gender |  |  |  |  |  |
| Male | 1.04 (0.70-1.54) | 1.00 | 0.86 (0.55-1.34) | 0.82 (0.49-1.38) | 1.04 (0.55-1.97) |
| Female | 1.27 (0.85-1.90) | 1.00 | 1.09 (0.73-1.63) | 1.33 (0.81-2.19) | 1.53 (0.83-2.79) |
| The *P* value of interaction between gender group and Weight change patterns | 0.3030 | - | 0.3720 | 0.0730 | 0.2072 |
| **Prevalence of heart attack** |  |  |  |  |  |
| Stratified by age , years |  |  |  |  |  |
| ≤65 | 1.43 (1.01-2.05) | 1.00 | 0.87 (0.58-1.29) | 1.12 (0.75-1.66) | 1.04 (0.66-1.65) |
| ＞65 | 1.11 (0.85-1.46) | 1.00 | 1.13 (0.85-1.52) | 1.09 (0.71-1.66) | 1.60 (0.78-3.28) |
| The *P* value of interaction between age group and Weight change patterns | 0.1350 | - | 0.3302 | 0.7057 | 0.4048 |
| Stratified by gender |  |  |  |  |  |
| Male | 1.29 (0.99-1.68) | 1.00 | 0.95 (0.70-1.29) | 0.93 (0.65-1.33) | 0.91 (0.63-1.30) |
| Female | 1.11 (0.81-1.53) | 1.00 | 1.09 (0.75-1.57) | 1.39 (0.95-2.03) | 1.59 (0.87-2.90) |
| The *P* value of interaction between gender group and Weight change patterns | 0.6098 | - | 0.4199 | 0.0388 | 0.0125 |
| **Prevalence of stroke** |  |  |  |  |  |
| Stratified by age , years |  |  |  |  |  |
| ≤65 | 2.44 (1.67-3.56) | 1.00 | 1.17 (0.82-1.68) | 2.20 (1.43-3.39) | 1.52 (0.95-2.44) |
| ＞65 | 1.28 (0.99-1.67) | 1.00 | 0.93 (0.64-1.33) | 1.18 (0.81-1.73) | 1.20 (0.70-2.07) |
| The *P* value of interaction between age group and Weight change patterns | 0.0034 | - | 0.3050 | 0.0134 | 0.2576 |
| Stratified by gender |  |  |  |  |  |
| Male | 1.64 (1.19-2.27) | 1.00 | 0.93 (0.61-1.41) | 1.63 (1.01-2.64) | 1.54 (0.83-2.83) |
| Female | 1.60 (1.23-2.07) | 1.00 | 0.98 (0.72-1.34) | 1.46 (1.04-2.06) | 1.09 (0.74-1.59) |
| The *P* value of interaction between gender group and Weight change patterns | 0.8968 | - | 0.6409 | 0.9278 | 0.6290 |

Abbreviations: 95% CI, 95% Confidence Interval; CVD, Cardiovascular diseases.

*All the estimated values have taken into account the complex sampling designs. Among 35998 participants, a total of 1512, 964, and 1001 participants were excluded in analyses from age 25 years to 10 years before baseline, age 25 years to baseline, and 10 years before baseline to baseline, respectively, owing to missing values of body mass index at both times. Model adjusted for age, gender, race/ethnicity, educational level, income level, marital status, alcohol consumption, smoking status, family history of diabetes, family history of heart disease, moderate exercise status and general health condition.

**Table S6 | Hazard ratios (95% CIs) of CVD mortality with absolute weight change groups***

|  | **Weight change patterns** | | | | |
| --- | --- | --- | --- | --- | --- |
|  | **Weight loss ≥2.5 kg** | **Weight change within 2.5 kg** | **Weight gain ≥2 .5 kg and <10.0 kg** | **Weight gain ≥10 kg and <20 kg** | **Weight gain ≥20 kg** |
| **From age 25 years to 10 years before baseline** |  |  |  |  |  |
| Cardiovascular diseases |  |  |  |  |  |
| No of deaths/person years | 146/16626.17 | 694/91488.00 | 533/96453.25 | 511/69885.58 | 430/48076.08 |
| Age adjusted mortality rate† | 10.02 (7.27-12.78) | 9.60 (8.44-10.77) | 7.26 (6.28-8.23) | 7.65 (6.51-8.78) | 8.76 (7.30-10.23) |
| Model 1 | 1.02 (0.86-1.22) | 1.00 | 0.75 (0.67-0.84) | 0.80 (0.71-0.89) | 0.97 (0.86-1.09) |
| Model 2 | 0.92 (0.66-1.28) | 1.00 | 0.85 (0.71-1.03) | 0.84 (0.69-1.02) | 0.97 (0.80-1.18) |
| Model 3 | 0.91 (0.65-1.27) | 1.00 | 0.85 (0.70-1.03) | 0.80 (0.66-0.98) | 0.95 (0.78-1.16) |
| **From age 25 years to baseline** |  |  |  |  |  |
| Cardiovascular diseases |  |  |  |  |  |
| No of deaths/person years | 356/24239.33 | 497/53588.67 | 446/70554.50 | 472/83917.42 | 543/90229.17 |
| Age adjusted mortality rate† | 13.87 (11.27-16.47) | 9.44 (8.07-10.82) | 7.66 (6.50-8.83) | 6.66 (5.67-7.64) | 7.72 (6.65-8.80) |
| Model 1 | 1.40 (1.22-1.61) | 1.00 | 0.79 (0.70-0.90) | 0.73 (0.64-0.83) | 0.89 (0.79-1.01) |
| Model 2 | 1.33 (1.05-1.69) | 1.00 | 0.90 (0.73-1.12) | 0.73 (0.59-0.90) | 0.88 (0.71-1.08) |
| Model 3 | 1.32 (1.03-1.67) | 1.00 | 0.87 (0.70-1.09) | 0.72 (0.58-0.89) | 0.85 (0.69-1.05) |
| **From 10 years before baseline to baseline** |  |  |  |  |  |
| Cardiovascular diseases |  |  |  |  |  |
| No of deaths/person years | 777/62950.17 | 783/94370.42 | 434/92329.17 | 194/48325.08 | 126/24554.25 |
| Age adjusted mortality rate† | 11.11 (9.72-12.5) | 8.00 (7.05-8.95) | 6.21 (5.26-7.16) | 6.96 (5.35-8.57) | 10.95 (7.61-14.28) |
| Model 1 | 1.43 (1.29-1.58) | 1.00 | 0.84 (0.75-0.94) | 1.02 (0.87-1.20) | 1.68 (1.38-2.04) |
| Model 2 | 1.40 (1.18-1.65) | 1.00 | 0.80 (0.66-0.98) | 1.01 (0.78-1.32) | 1.50 (1.09-2.07) |
| Model 3 | 1.40 (1.19-1.65) | 1.00 | 0.78 (0.63-0.96) | 1.03 (0.80-1.34) | 1.47 (1.07-2.04) |

Abbreviations: 95% CI, 95% Confidence Interval; CVD, Cardiovascular diseases.

*All the estimated values have taken into account the complex sampling designs. Among 35998 participants, a total of 1512, 964, and 1001 participants were excluded in analyses from age 25 years to 10 years before baseline, age 25 years to baseline, and 10 years before baseline to baseline, respectively, owing to missing values of body mass index at both times. Model 1 adjusted for baseline age, gender and race/ethnicity. Model 2 further adjusted for educational level, income level, marital status, alcohol consumption, smoking status, family history of diabetes, and family history of heart disease. Model 3 further adjusted for baseline moderate exercise status and general health condition.

†Mortality rates per 1000 person years, directly standardised to age distribution of entire study population.

**Table S7 | Hazard Ratios (95% CIs) of CVD mortality with weight change stratified by age and gender***

|  | **Weight change patterns** | | | | |
| --- | --- | --- | --- | --- | --- |
|  | **Stable normal** | **Maximum overweight** | **Obese-non-obese** | **Non-obese-obese** | **Stable obese** |
| **From age 25 years to 10 years before baseline** |  |  |  |  |  |
| Stratified by age , years |  |  |  |  |  |
| ≤65 | 1.00 | 0.96 (0.66-1.40) | 2.84 (1.13-7.12) | 1.75 (1.21-2.53) | 3.50 (2.20-5.57) |
| ＞65 | 1.00 | 0.96 (0.80-1.16) | 0.88 (0.33-2.37) | 1.07 (0.87-1.33) | 1.32 (0.82-2.13) |
| The *P* value of interaction between age group and Weight change patterns | - | 0.8558 | 0.0886 | 0.0269 | 0.0037 |
| Stratified by gender |  |  |  |  |  |
| Male | 1.00 | 1.05 (0.84-1.31) | 0.62 (0.20-1.94) | 1.31 (1.02-1.69) | 2.00 (1.34-3.00) |
| Female | 1.00 | 0.79 (0.61-1.04) | 3.81 (1.67-8.69) | 0.95 (0.72-1.25) | 1.65 (0.98-2.75) |
| The *P* value of interaction between gender group and Weight change patterns | - | 0.0826 | 0.0124 | 0.0532 | 0.4450 |
| **From age 25 years to baseline** |  |  |  |  |  |
| Stratified by age , years |  |  |  |  |  |
| ≤65 | 1.00 | 1.03 (0.68-1.57) | 5.51 (2.86-10.63) | 1.47 (0.99-2.18) | 2.83 (1.64-4.88) |
| ＞65 | 1.00 | 1.04 (0.86-1.26) | 1.28 (0.65-2.50) | 0.95 (0.76-1.18) | 1.18 (0.68-2.04) |
| The *P* value of interaction between age group and Weight change patterns | - | 0.9921 | 0.0018 | 0.0349 | 0.0199 |
| Stratified by gender |  |  |  |  |  |
| Male | 1.00 | 1.13 (0.90-1.41) | 1.74 (0.98-3.09) | 1.13 (0.87-1.46) | 1.60 (0.99-2.59) |
| Female | 1.00 | 0.87 (0.66-1.13) | 3.43 (1.66-7.07) | 0.85 (0.64-1.13) | 1.52 (0.88-2.64) |
| The *P* value of interaction between gender group and Weight change patterns | - | 0.0999 | 0.1831 | 0.0744 | 0.7672 |
| **From 10 years before baseline to baseline** |  |  |  |  |  |
| Stratified by age , years |  |  |  |  |  |
| ≤65 | 1.00 | 0.94 (0.60-1.46) | 3.08 (1.80-5.27) | 1.30 (0.81-2.10) | 1.94 (1.27-2.97) |
| ＞65 | 1.00 | 1.02 (0.83-1.25) | 1.40 (1.05-1.87) | 0.98 (0.72-1.34) | 0.99 (0.77-1.29) |
| The *P* value of interaction between age group and Weight change patterns | - | 0.7970 | 0.0106 | 0.2340 | 0.0048 |
| Stratified by gender |  |  |  |  |  |
| Male | 1.00 | 1.07 (0.83-1.36) | 1.88 (1.36-2.62) | 1.13 (0.80-1.60) | 1.27 (0.95-1.69) |

**Continuous Table S7 | Hazard Ratios (95% CIs) of CVD mortality with weight change stratified by age and gender***

|  | **Weight change patterns** | | | | |
| --- | --- | --- | --- | --- | --- |
|  | **Stable normal** | **Maximum overweight** | **Obese-non-obese** | **Non-obese-obese** | **Stable obese** |
| Female | 1.00 | 0.87 (0.65-1.15) | 1.19 (0.78-1.80) | 0.84 (0.58-1.22) | 0.94 (0.68-1.29) |
| The *P* value of interaction between gender group and Weight change patterns | - | 0.1894 | 0.0579 | 0.1582 | 0.0970 |

Abbreviations: 95% CI, 95% Confidence Interval; CVD, Cardiovascular diseases.

*All the estimated values have taken into account the complex sampling designs. Among 35998 participants, a total of 1512, 964, and 1001 participants were excluded in analyses from age 25 years to 10 years before baseline, age 25 years to baseline, and 10 years before baseline to baseline, respectively, owing to missing values of body mass index at both times. Model adjusted for age, gender, race/ethnicity, educational level, income level, marital status, alcohol consumption, smoking status, family history of diabetes, family history of heart disease, moderate exercise status and general health condition.

**Table S8 | Hazard Ratios (95% CIs) of CVD mortality with absolute weight change stratified by age, gender***

|  | **Absolute weight change groups** | | | | |
| --- | --- | --- | --- | --- | --- |
|  | **Weight loss ≥2.5 kg** | **Weight change within 2.5 kg** | **Weight gain ≥2 .5 kg and <10.0 kg** | **Weight gain ≥10 kg and <20 kg** | **Weight gain ≥20 kg** |
| **From age 25 years to 10 years before baseline** |  |  |  |  |  |
| Stratified by age , years |  |  |  |  |  |
| ≤65 | 1.49 (0.83-2.69) | 1.00 | 1.17 (0.78-1.75) | 1.20 (0.79-1.85) | 1.78 (1.17-2.69) |
| ＞65 | 0.77 (0.52-1.15) | 1.00 | 0.80 (0.65-0.99) | 0.75 (0.60-0.94) | 0.88 (0.70-1.10) |
| The *P* value of interaction between age group and Weight change patterns | 0.0512 | - | 0.2565 | 0.1834 | 0.0202 |
| Stratified by gender |  |  |  |  |  |
| Male | 0.96 (0.63-1.46) | 1.00 | 1.06 (0.83-1.37) | 1.10 (0.84-1.43) | 1.18 (0.91-1.54) |
| Female | 0.93 (0.56-1.55) | 1.00 | 0.64 (0.48-0.87) | 0.53 (0.39-0.73) | 0.71 (0.52-0.96) |
| The *P* value of interaction between gender group and Weight change patterns | 0.9054 | - | 0.0071 | 0.0003 | 0.0100 |
| **From age 25 years-baseline** |  |  |  |  |  |
| Stratified by age , years |  |  |  |  |  |
| ≤65 | 2.28 (1.38-3.78) | 1.00 | 1.04 (0.62-1.74) | 0.91 (0.55-1.50) | 1.22 (0.77-1.92) |
| ＞65 | 1.13 (0.86-1.48) | 1.00 | 0.84 (0.66-1.07) | 0.70 (0.55-0.90) | 0.83 (0.65-1.06) |
| The *P* value of interaction between age group and Weight change patterns | 0.0045 | - | 0.5131 | 0.4293 | 0.1111 |
| Stratified by gender |  |  |  |  |  |
| Male | 1.44 (1.06-1.97) | 1.00 | 1.08 (0.82-1.44) | 0.84 (0.63-1.13) | 1.07 (0.81-1.42) |
| Female | 1.21 (0.83-1.75) | 1.00 | 0.64 (0.45-0.91) | 0.59 (0.42-0.82) | 0.63 (0.46-0.86) |
| The *P* value of interaction between gender group and Weight change patterns | 0.4117 | - | 0.0143 | 0.0768 | 0.0067 |
| **From 10 years before baseline to baseline** |  |  |  |  |  |
| Stratified by age , years |  |  |  |  |  |
| ≤65 | 1.84 (1.25-2.71) | 1.00 | 0.82 (0.52-1.27) | 1.13 (0.70-1.81) | 1.45 (0.89-2.34) |
| ＞65 | 1.32 (1.10-1.59) | 1.00 | 0.80 (0.63-1.02) | 1.06 (0.77-1.46) | 1.58 (1.01-2.48) |
| The *P* value of interaction between age group and Weight change patterns | 0.0524 | - | 0.8596 | 0.4471 | 0.5037 |

**Continuous Table S8 | Hazard Ratios (95% CIs) of CVD mortality with absolute weight change stratified by age, gender***

|  | **Absolute weight change groups** | | | | |
| --- | --- | --- | --- | --- | --- |
|  | **Weight loss ≥2.5 kg** | **Weight change within 2.5 kg** | **Weight gain ≥2 .5 kg and <10.0 kg** | **Weight gain ≥10 kg and <20 kg** | **Weight gain ≥20 kg** |
| Stratified by gender |  |  |  |  |  |
| Male | 1.57 (1.26-1.95) | 1.00 | 0.98 (0.75-1.28) | 0.96 (0.66-1.39) | 1.78 (1.17-2.70) |
| Female | 1.16 (0.89-1.51) | 1.00 | 0.54 (0.38-0.77) | 1.07 (0.74-1.55) | 1.22 (0.76-1.95) |
| The *P* value of interaction between gender group and Weight change patterns | 0.0828 | - | 0.0052 | 0.7859 | 0.1318 |

Abbreviations: 95% CI, 95% Confidence Interval; CVD, Cardiovascular diseases.

*All the estimated values have taken into account the complex sampling designs. Among 35998 participants, a total of 1512, 964, and 1001 participants were excluded in analyses from age 25 years to 10 years before baseline, age 25 years to baseline, and 10 years before baseline to baseline, respectively, owing to missing values of body mass index at both times. Model adjusted for age, gender, race/ethnicity, educational level, income level, marital status, alcohol consumption, smoking status, family history of diabetes, family history of heart disease, moderate exercise status and general health condition.
